# Cervical Cancer Stem-Like Cell Transcriptome Profiles Predict Response to Chemoradiotherapy

**DOI:** 10.3389/fonc.2021.639339

**Published:** 2021-05-07

**Authors:** Luciana W. Zuccherato, Christina M. T. Machado, Wagner C. S. Magalhães, Patrícia R. Martins, Larissa S. Campos, Letícia C. Braga, Andrea Teixeira-Carvalho, Olindo A. Martins-Filho, Telma M. R. F. Franco, Sálua O. C. Paula, Israel Tojal da Silva, Rodrigo Drummond, Kenneth J. Gollob, Paulo Guilherme O. Salles

**Affiliations:** ^1^ Núcleo de Ensino e Pesquisa - Instituto Mário Penna, Belo Horizonte, Brazil; ^2^ Instituto René Rachou - Fiocruz, Belo Horizonte, Brazil; ^3^ Instituto Mário Penna, Belo Horizonte, Brazil; ^4^ International Research Center, A.C. Camargo Cancer Center, São Paulo, Brazil; ^5^ Translational Immuno-Oncology Laboratory, International Research Center, A.C. Camargo Cancer Center, São Paulo, Brazil

**Keywords:** stem-like cells, cervical cancer, low input RNA sequencing, biomarkers, chemoradioresistance

## Abstract

Cervical cancer (CC) represents a major global health issue, particularly impacting women from resource constrained regions worldwide. Treatment refractoriness to standard chemoradiotheraphy has identified cancer stem cells as critical coordinators behind the biological mechanisms of resistance, contributing to CC recurrence. In this work, we evaluated differential gene expression in cervical cancer stem-like cells (CCSC) as biomarkers related to intrinsic chemoradioresistance in CC. A total of 31 patients with locally advanced CC and referred to Mário Penna Institute (Belo Horizonte, Brazil) from August 2017 to May 2018 were recruited for the study. Fluorescence-activated cell sorting was used to enrich CD34+/CD45- CCSC from tumor biopsies. Transcriptome was performed using ultra-low input RNA sequencing and differentially expressed genes (DEGs) using Log2 fold differences and adjusted p-value < 0.05 were determined. The analysis returned 1050 DEGs when comparing the Non-Responder (NR) (n=10) and Responder (R) (n=21) groups to chemoradiotherapy. These included a wide-ranging pattern of underexpressed coding genes in the NR *vs.* R patients and a panel of lncRNAs and miRNAs with implications for CC tumorigenesis. A panel of biomarkers was selected using the rank-based AUC (Area Under the ROC Curve) and pAUC (partial AUC) measurements for diagnostic sensitivity and specificity. Genes overlapping between the 21 highest AUC and pAUC loci revealed seven genes with a strong capacity for identifying NR *vs.* R patients (*ILF2, RBM22P2, ACO16722.1, AL360175.1* and *AC092354.1*), of which four also returned significant survival Hazard Ratios. This study identifies DEG signatures that provide potential biomarkers in CC prognosis and treatment outcome, as well as identifies potential alternative targets for cancer therapy.

## Introduction

Cervical Cancer (CC) is the second most frequent cancer and the fourth leading cause of cancer deaths in women worldwide. The Global Cancer Observatory (GLOBOCAN) estimates the burden of CC incidence in 2018 reached almost 570,000 women and a mortality rate of 54.6% (311,365 patients). Approximately 85% of cases occur in low- and middle-income countries (LMICs), and are predominantly diagnosed in advanced stages (1). Despite the scientific advances in primary and secondary prevention (vaccine, HPV screening and precancerous lesions treatment, respectively), CC continues to be a major global health challenge. Impressively, around 50% of patients with CC died as a consequence of treatment failure and other cancer- related complications. This dismal scenario is also reflected in South American patients (2), and highlights the importance of developing novel therapeutic approaches for CC and achieving a more personalized medical care.

The heterogeneous cellular composition of tumors leads to extreme genetic and epigenetic diversity and thereby produces a plethora of biological factors that can result in a poor prognosis and low survival rates (3). Cancer Stem Cells (CSCs) are a pivotal participant in these processes. In normal tissues, stem cells are generally defined by a controlled self-renewal feature, with the ability to produce both specialized and undifferentiated tissue-maintaining cells. Conversely, CSCs can display perturbed growth properties, leading to cancer initiation, chemoresistance, and metastasis (4). Tumor heterogeneity can therefore be supported by the CSC paradigm, where a subset of cells, organized into hierarchical structures based on differentiation capacity, drive malignancy and therapeutic refractoriness (5).

Given the importance of CSCs in cancer pathogenicity, it is not unexpected that many studies have sought to uncover the molecular pathways related to stemness. As observed in normal stem cells, CSCs exhibit genetic markers and pathways typically associated with proliferation (6). Aberrant expression of transcription factors *SOX2, NANOG, OCT3/4*, c-Myc (7), and disruption of Wnt/β-catenin, Hedgehog, Notch and PI3K/AKT/mTOR signaling pathways are representative hallmarks that sustain the stem cell phenotype in CSC and support therapy resistance (8). Alternatively, mutations encompassing the tumor suppressor genes *TP53, PTEN*, and INK4A-ARF locus have been implicated in stem cell DNA damage pathways and self-renewal deregulation (6).

Given the importance of CSCs in the tumorigenic process of solid tumors, we aimed to study differential expressed genes (DEG) in cervical cancer stem-like cells (CCSC) from tumor biopsies taken before treatment began in a cohort of CC patients that responded or not to chemoradiotherapy. The analysis of DEGs from sorted CCSC between responder *vs.* non-responder patients points to a set of potential biomarkers for prediction of response to chemoradiotherapy, as well as offering new insights into the potential role of CCSCs in therapy failure.

## Materials and Methods

### Patient Recruitment and Sample Selection

A total of 31 patients with CC and referred to Mário Penna Institute (Belo Horizonte, Brazil) from August 2017 to May 2018 were recruited to the study. Inclusion criteria was histopathological diagnosis of Squamous cell carcinoma or Adenocarcinoma, no previous history of cancer or immune diseases. Cervical biopsies (FIGO stages II and III) were collected after participation agreement and signed the consent form. All patients were further submitted to radiotherapy concomitantly with chemotherapy, and clinical data were collected from medical records. The study was approved by the local Institutional Review Board (Number 1.583.784).

Patients were screened as Responders (R) or Non-Responders (NR) based on Gynecologic Oncology parameters of absence/persistence of cervical lesions 8 months after the chemoradiotherapy. Clinical examination (vaginal, pelvic, abdominal), laboratorial analysis (cytology, new biopsy) and imaging (Ultrasound, Computer Tomography and Magnetic Resonance Imaging, when available) were assigned up to 8 months after treatment. Patients were considered R when cervical lesions were undetected after chemoradiotherapy. Patients with persistence of lesions after the treatment (partial response, tumor progression or stable disease) were classified as NR.

### Fluorescence-Activated Cell Sorting

Fluorescence-activated cell sorting (FACS) was used to isolate enriched CCSCs from a complex mixture of tumor cells based on their light scatter and fluorescent staining profiles. CC tissue fragments (5mm) from the 31 patients were fragmented using Med Machine^®^ according to manufacturer’s instructions (BD-Biosciences). Cell suspension containing CCSCs was frozen in 20% HES cryoprotective solution (100 mL anhydrous glucose 1.7 g/L; Na(+1) 140 mEq/L; Cl(-1) 98 mEq/L; K(+1) 5 mEq/L; Mg (+2) 3 mEq/L; Gluconate 23 mEq/L; Acetate 27 mEq/L) and stored in liquid nitrogen until use. Considering the scarcity of CCSCs, two monoclonal antibodies, cell surface markers CD45 (APCH7 Clone 2D1) and CD34 (PE Clone 563) were used in the cell suspension for further FACS selection.

Cell concentration was increased to 5 x10^6^ cells/mL. CCSCs-enriched subpopulations were isolated using FACSAria^®^ flow-sorter (BD-Biosciences). Yield mode was performed at 45 psi with 85-μm nozzle at a frequency of ∼51 kHz. Two fluorescence channels were analyzed (APC-H7 and PE). Cells were distinguished from debris in the sample by distinct FSC values, since debris can be identified as particles with lowest FSC values. Sorting of CCSCs was performed using a gate containing the CD45-/CD34+ population to eliminate contamination with hematopoietic stem cells, thereby enriching for CCSC-like cells (**Supplementary Figure 1**). Cells were considered positive above 10^2^ for each parameter based on negative populations defined by their autofluorescence. The CCSCs were sorted into cytometry tubes containing 1 μL of Lysis Solution (Lysis buffer and RNAse inhibitor from Takara^®^ Kit Smart Seq V4 RNA) for genomic library construction and sequencing.

### cDNA Synthesis

SMART-Seq v4 Ultra-low Input RNA Kit for Sequencing (Takara Bio USA, CA) was used to generate the full-length cDNA from the selected sorted cells following the manufacturer’s instructions. Reactions with positive (Control Total RNA, provided by SMART-Seq v4 Ultra-low Input RNA Kit) and negative controls were carried out for quality control. Successful cDNA synthesis and amplification were considered when an Agilent High Sensitivity DNA Chip run on the Agilent Bioanalyzer 2100 (Agilent, CA) showed an electropherogram with a distinct peak spanning from 400 bp to 10,000 bp, and cDNA concentration ≥ 0.3 ng/μl detected with Qubit™ dsDNA HS Assay Kit on a Qubit 3 Fluorometer (Thermo Fisher Scientific, MA). Purified cDNAs were stored at -20°C for further processing.

### Sequencing Libraries

Library preparation of suitable cDNAs were performed using Nextera^®^ XT Library Prep Kit (Illumina, CA) with Nextera^®^ XT Index Kit V2 Set A (Illumina, CA). Samples were normalized to 40 pg/μl for a total 200 pg input of amplified cDNA. The protocol was performed as described by the manufacturer. Libraries were purified with 0.6 x bead ratio using Agencourt AMPure beads XP (Beckman Coulter, IN) and eluted in 52.5 μl of elution buffer. Quality parameters as size (440 bp average) and concentration (1.03 ng/μl average) were measured using High Sensitivity D1000 ScreenTape and reagents run on 2200 TapeStation System (Agilent, CA) and Qubit™ dsDNA HS Assay Kit on a Qubit 3 Fluorometer (Thermo Fisher Scientific, MA), respectively. Good quality libraries were normalized to 1 nM. Thirteen samples were pooled to further perform 101 cycles of single read sequencing using a NextSeq^®^ 500/550 High Output Kit v2 (150 cycles) and NextSeq^®^ 550 sequencer (Illumina, CA). Sixty-two libraries with more than 20 million reads each were considered for analysis.

### RNA-Seq Data Analysis

Sequencing quality control and adapters were analyzed in the FastQ files using FastQC version 0.11.9. Trimming of the adaptor content and overrepresented sequences was performed using Trimmomatic (9). Quality check using FastQC was also performed on the trimmed sequences. Reads from the fastq files were aligned to the human reference (build GRCH38 and annotation file Homo_sapiens.GRCh38.83.gtf) using the 2-Pass protocol from the STAR software (10). The resulting alignment file was compressed, indexed and name-sorted using the samtools (version 0.1.19- 44428cd). The count table was generated using GeneCounts mode from STAR. All steps are summarized in the **Supplementary Figure 2**; command lines and Pearl scripts of the workflow are available upon request.

### Differentially Expressed Genes (DEG) Analysis and Biomarkers Selection

Count data were imported to R software (11) and DESeq2 package (12) were used to perform differential expression analysis. Benjamini and Hochberg procedure, implemented in DESeq2, were used for p-values adjustment and a False Discovery Rate (FDR) cutoff of 0.05 was applied for assigning a given gene as a DEG between the groups NR and R. The panel of biomarkers were proposed based on the following criteria: (i) 22 highest values of AUC (area under the curve), (ii) 21 highest values of pAUC (partial area under the ROC curve statistic - (pAUC - 0.10) (13) and lastly, (iii) the common loci between the two selection criteria. All statistics were calculated using the R package “genefilter”.

### Stemness Genes

The transcript abundance of stemness genes described in CC studies (**Supplementary Table 1**) across NR and R samples were compared by using Wilcoxon rank-sum test. Distribution of regularized log2 expression for statistically significant genes (p<0.05) were displayed as boxplots.

### Screening microRNA Targets

To assess the putative regulation of target genes of miRNA, we downloaded all predictions for representative transcripts performed by targetScan - v72 (14). Only both miRNA and targets differentially expressed (adjusted p-value<0.05) with opposite regulation among NR and R according to Rfold change values were used in the further analysis.

### Gene Enrichment Analysis and Networks

GO (Gene Ontology) function enrichment analyses of the 1050 DEGs and gene targets of miRNA were performed through clusterProfiler package (15). STRING (https://string-db.org) was employed to predict potential interactions between *ILF2* and other proteins.

### Statistical Analysis

Survival outcomes and the hazard ratio for disease progression or death were assessed *via* Kaplan-Meier methods and compared using Log-rank tests, using GraphPad Prism 5.0 (GraphPad Software Inc., La Jolla, Ca, USA). Forest plots represent the hazard ratio analysis of gene expression, obtained using a univariate Cox regression model in R (version 3.6.3, (Team, 2020 #3817); https://www.R-project.org, within survival package (version 3.2-3, https://CRAN.R-project.org/package=survival). Genes were selected by pAUC analysis.

## Results

### Clinicopathological Characteristics of the Cohort

**Figure 1:**
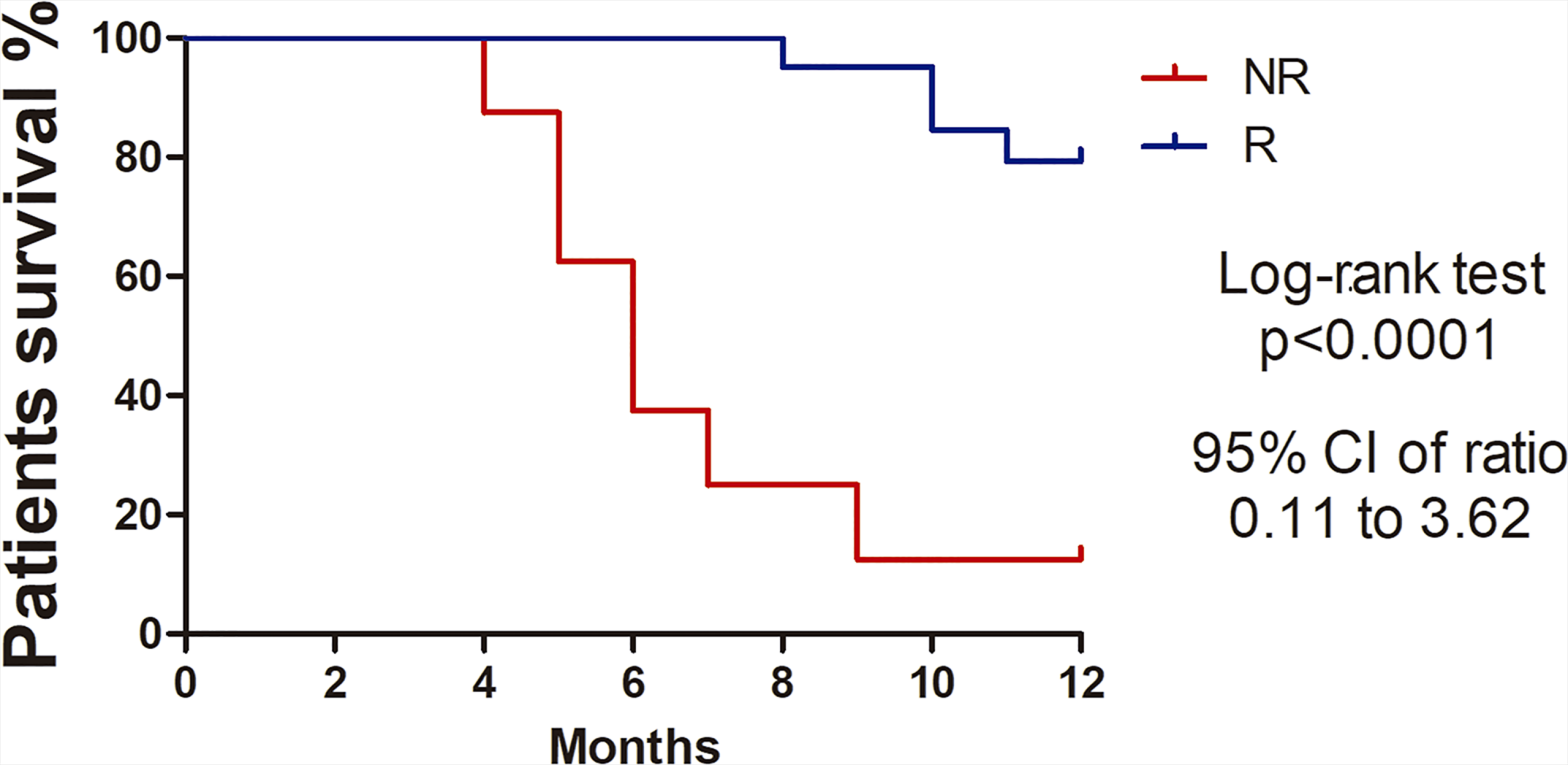
Kaplan-Meier survival curves showing higher percentage survival in Responder patients with cervical cancer. Log-rank test (Chi square 22.34, p<0.0001). Hazard ratio 0.64 (95%, CI 0.11-3.62).

Samples from 31 women with an average age of 52.3 ± 16.8 years-old (range 24-82) were submitted to NGS analysis. Approximately 96% of patients had squamous cell carcinoma, only one patient had adenocarcinoma, stages FIGO II-B (48.3%) and III-B (48.3%). The mean tumor size was 6.8 cm. Most patients had bilateral parametrial (71%) and vaginal involvement (90%) and tumor lesions were moderately (42%) or poorly differentiated (42%). The overall response was evaluated 8 months after the end of treatment and 21 patients were classified as Responders (R) and 10 as Non-responders (NR). Clinical information from the patients and histopathological analysis are summarized in **Supplementary Table 2**. Kaplan-Meier curves highlighted the poorer survival outcomes of NR patients with 12% survival at 12 months, whereas the R group showed 79% survival (Hazard Ratio [HR]: 6.44 95% CI: 0.11–3.62, p<0.0001). (**Figure 1**).

### Differential Expressed Genes (DEGs) in Cervical Cancer Stem-Like Cells (CCSC)

**Figure 2:**
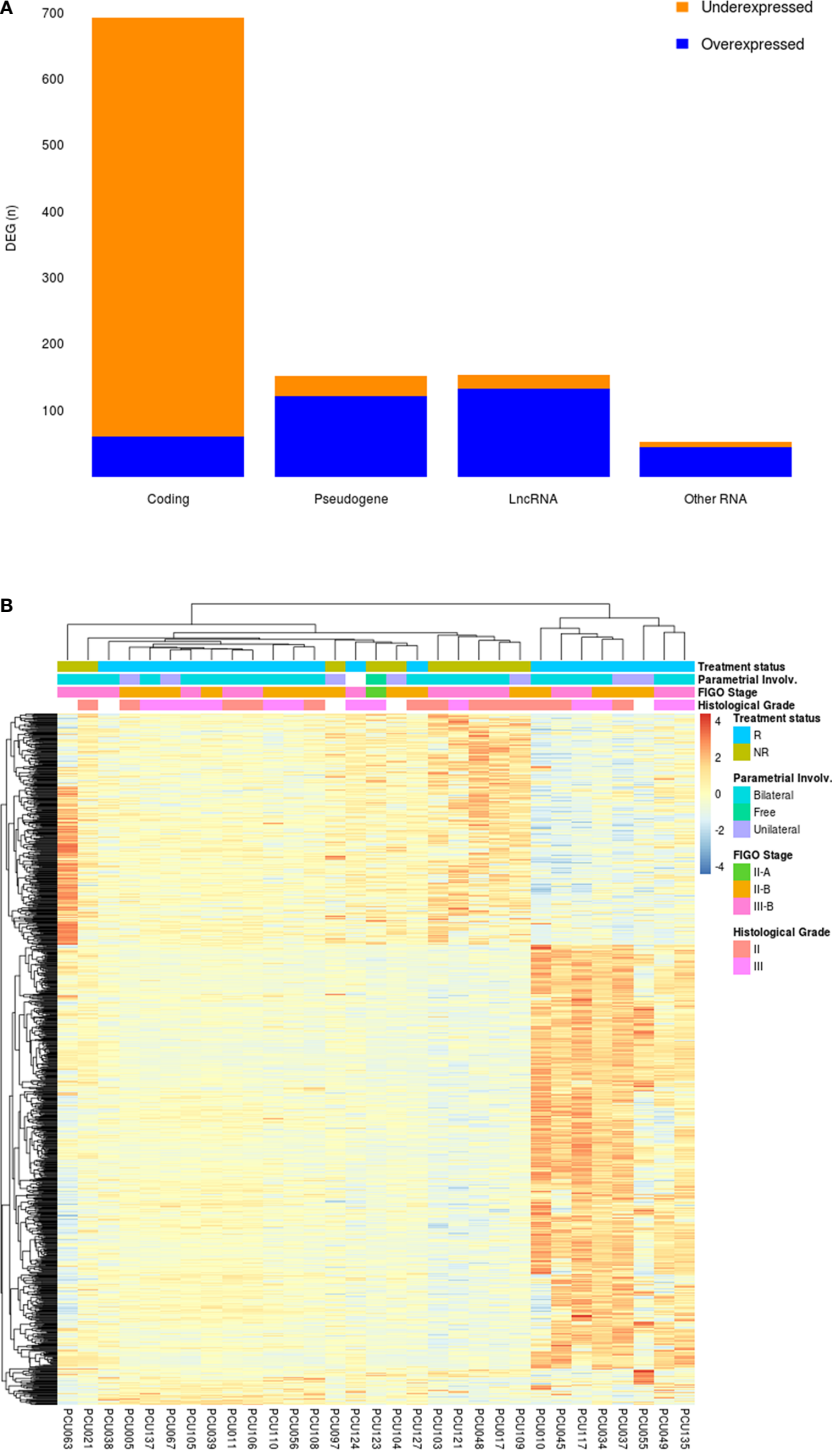
**(A)** Frequency of DEGs from cervical cancer stem-like cells in Non-Responder (n=10) compared to Responder patients (n=21). From the 1050 DEGs (adjusted p-value < 0.05), 694 protein coding, 150 pseudogenes, 154 long non-coding RNAs (LncRNA) and 52 transcripts classified as “Other RNA” (snoRNA, miRNA, miscRNA, TEC, snRNA and mitochondrial rRNA) were identified. Orange and blue colors represent the proportion of under and overexpressed DEG transcripts in each category, respectively. **(B)** Heatmap showing 1050 differential expressed genes (adjusted p-value < 0.05) in cervical cancer stem-like cells. Responder patients (R; n=21) are represented in blue and Non-Responders (NR; n=10) are colored in green. Clinical features of Parametrial involvement, FIGO Stages and Histological grades are detailed in the figure.

DEGs between NR and R patients were analyzed using an adjusted p value of < 0.05. This analysis returned 1050 differentially expressed transcripts. Ninety-one percent (91%; 633/694) of the coding genes were under expressed in NR patients; conversely, 86% of long non-coding RNAs (lncRNAs; 132/154) and 81% of pseudogenes (121/150) were overexpressed in the NR group. The snoRNA (Small nucleolar RNA), Mt_tRNA (Mitochondrial transfer RNA), misc_RNA (miscellaneous RNA), miRNA (microRNA), TEC (To be Experimentally Confirmed) and snRNA (Small nucleolar RNA) were grouped as “Other RNA” (n=52). The proportion of the DEGs and functional classes are detailed in **Figure 2A**.

An unsupervised clustering analysis of the 1050 DEGs revealed four main clusters related to the patients’ treatment outcome (NR *vs.* R) **(** **Figure 2B** **)**. A heterogeneous expression profile is apparent across the genes as exemplified by the heatmap. The patients segregated into clusters based on an expression pattern in agreement with the failure/success to chemoradiation treatment, with few exceptions. Clinicopathological characteristics of the patients, including pathologic parametrial involvement, FIGO stages and tumor pathological grades did not differentially segregate between the groups.

### Gene Ontology (GO) Classification and Functional Enrichment Analysis

**Figure 3:**
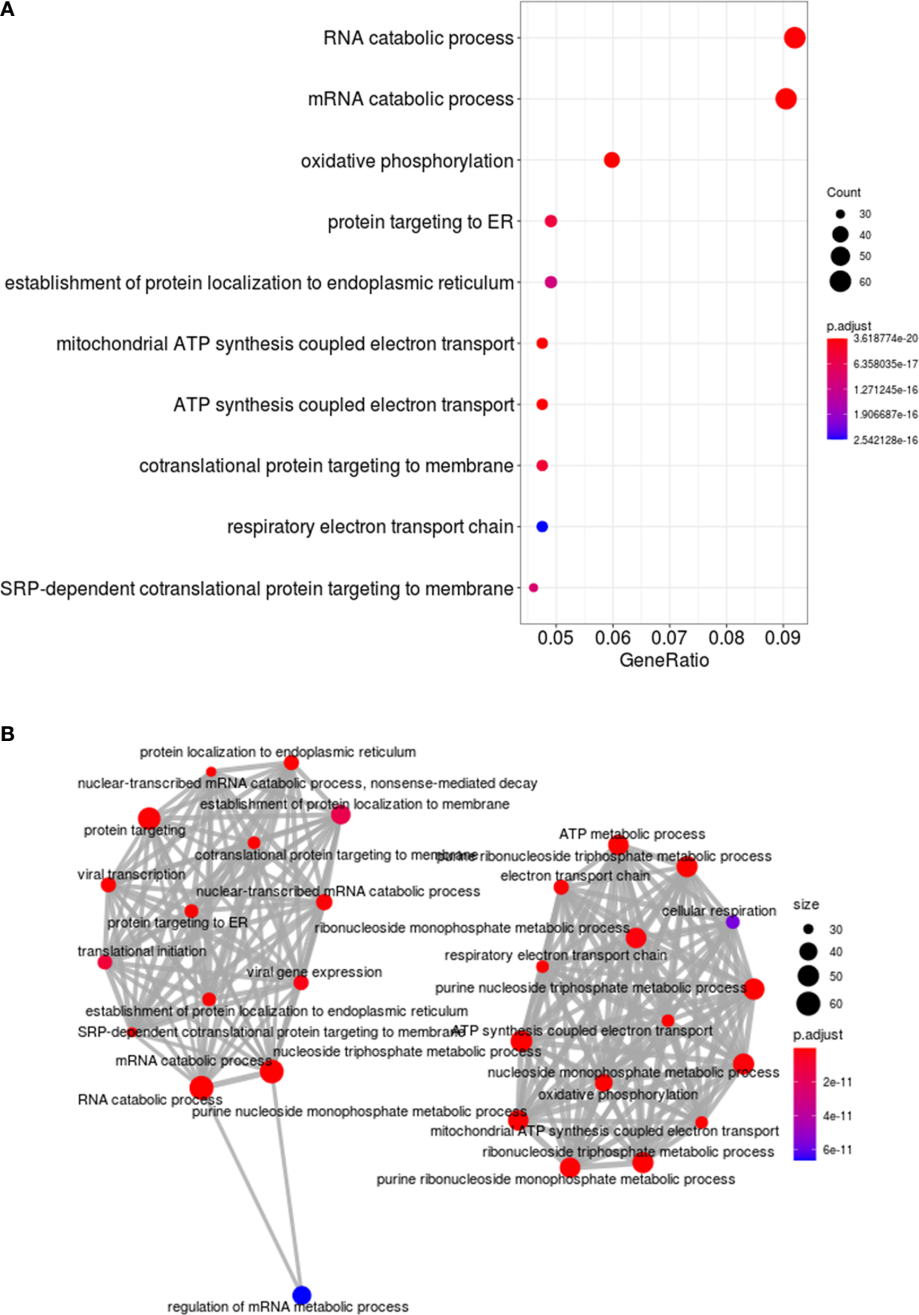
Functional enrichment analysis of DEGs in cervical cancer stem-like cells from Non-Responder (n=10) compared to Responder patients (n=21). **(A)** The top ten Biological process GO terms significantly enriched from clusterProfiler analysis of the 1050 DEGs. The dots are colored by the statistical significance of a GO term (lowest to highest p-values), and size represents the ratio of genes associated with the GO term and the total number of genes. **(B)** Enrichment map of the most significantly enriched Biological process terms. The GO terms are depicted as nodes, connected by overlapping gene sets. The node color and size correspond to statistical significance of a GO term enrichment and the number of genes in the set, respectively.

Analysis and visualization of enriched functional profiles from the 1050 DEGs was performed to explore gene-related biological processes using the clusterProfiler package. Overall, transcripts were statistically assigned to 171 GO terms with all downregulated in the NR group (**Supplementary Table 3**). Genes were enriched for transcriptional/translational processes, metabolism, and respiratory pathways (**Figure 3**). Specifically, the top gene ontology (GO) terms were related to “RNA catabolic process”, “Oxidative phosphorylation”, “Protein targeting to ER” and “Mitochondrial ATP synthesis” (**Figure 3A**). The enrichment map shows two main functional modules, clustering the GO terms related to nucleic acids/protein metabolism and energetic activity in disconnected networks (**Figure 3B**). The genes composing the two networks and their associations with the GO Biological Processes are shown in **Supplementary Figure 3**.

### Stemness Genes

**Figure 4:**
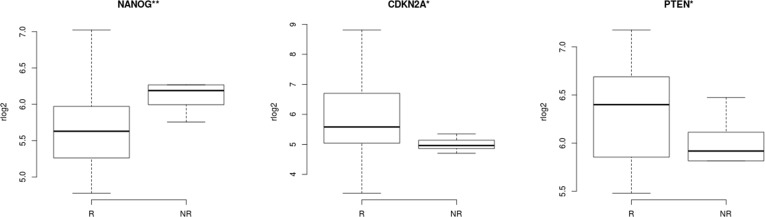
Correlation of stemness features in cervical cancer stem-like cells and response to therapy. CCSCs expression levels of genes related to stemness in cancer revealed three transcripts with statistically significant differences between Non-Responder (n=10) compared to Responder patients (n=21). *CDKN2A* (p=0.038), *NANOG* (p=0.006), *PTEN* (p=0.048), Wilcoxon rank-sum test. **<0.05 **<0.01.

The distribution of regularized gene expression levels (rlog) from 51 stemness genes described in CC studies and tumor suppressor genes related to stemness *TP53* and *PTEN* (**Supplementary Table 1**) was compared between NR and R patients. Wilcoxon rank-sum test revealed that CCSCs transcript levels of the genes *CDKN2A* (p=0.038) and *PTEN* (p=0.048) were decreased, while *NANOG* (p=0.006) was increased in the NR group. (**Figure 4**).

### Non-Coding RNAs and Epigenetic Findings in CCSC

The long-noncoding RNAs (lncRNAs) differentially expressed in NR and R (n=154) represent 14.7% of all transcripts. From those, we detected nine transcripts previously related to CC progression (**Table 1**) and 15 related to diverse cancers (**Supplementary Table 4**). From the small non-coding transcripts (snoRNA, Mt_tRNA, TEC, misc_RNA, miRNA and snRNA), we identified four microRNAs (MIR4278, MIR4422, MIR4779, MIR1268B) and one snoRNA (SNORA12) with aberrant expression previously associated with cancer pathogenesis (**Table 1**; **Supplementary Table 4**).

**Table 1:** Long non-coding RNAs (lncRNA) and small RNA (snoRNA*) differentially expressed in cervical cancer stem-like cells from Non-responders (NR) and Responders (R) related to cancer pathogenesis.

Gene	Log2FC	PADJ	Role in Cervical Cancer	Expression and cell types
***MIR205HG***	-2.75	5.00E-04	Modulates biological activities of CC cells targeting SRSF1 and regulating KRT17	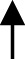 CaSki, MS751, HeLa and SiHa (16)
***MALAT1***	-2.09	2.00E-04	Predicts a poor prognosis of CC, promotes cancer cell growth and invasion/cisplatin resistance	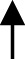 CC tissues, SiHa, ME-180, C4-I and C4-II (17); HeLa and C-33A (18)
***SNHG8***	-2.26	2.78E-02	Accelerates proliferation and inhibits apoptosis in HPV-induced CC	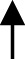 HeLa, SiHa, MS751, CaSki and C33-A (19)
***NEAT1***	-2.07	8.70E-03	Overexpression associated with radiosensitivity and CC poor prognosis	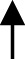 Non-sensitivity CC tissues, HeLa and SiHa (20)
***SNHG6***	-1.83	3.39E-02	Enhances the radio resistance and promotes growth of CC cells	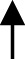 CC tissues, C-33A, SiHa, HeLa, CaSki, HT-3 (21)
***GAS5***	-1.78	1.06E-02	Increases proliferation and migration; low expression in chemoradioresistant CC tissues	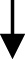 CC tissues, HeLa, SiHa (22, 23) metastatic lymph nodes, C-33A, Caski (22); 293T cells (23)
***NORAD***	-1.73	1.43E-02	Up regulated in CC tissues and cell lines	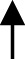 SiHa, HeLa, ME180, C-33A, CaSki (24)
***LINC01133***	-0.75	4.97E-02	Associated with CC progression and survival	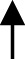 TCGA-cervical squamous cell carcinoma dataset (25)
SNORA12*	2.38	1.10E-02	Downregulated in CC	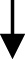 Cervical tumors, HeLa and SiHa (26)

Based on lncRNAs and small RNAs from public databases and literature, 9 transcripts associated with CC tumorigenesis were detected. Log2 fold change (Log2FC) values represent the difference in expression observed in NR when compared to R. (PADJ, Adjusted p-value <0.05; CC, cervical cancer).

### miRNA-Target Gene Pairs Analysis

**Figure 5:**
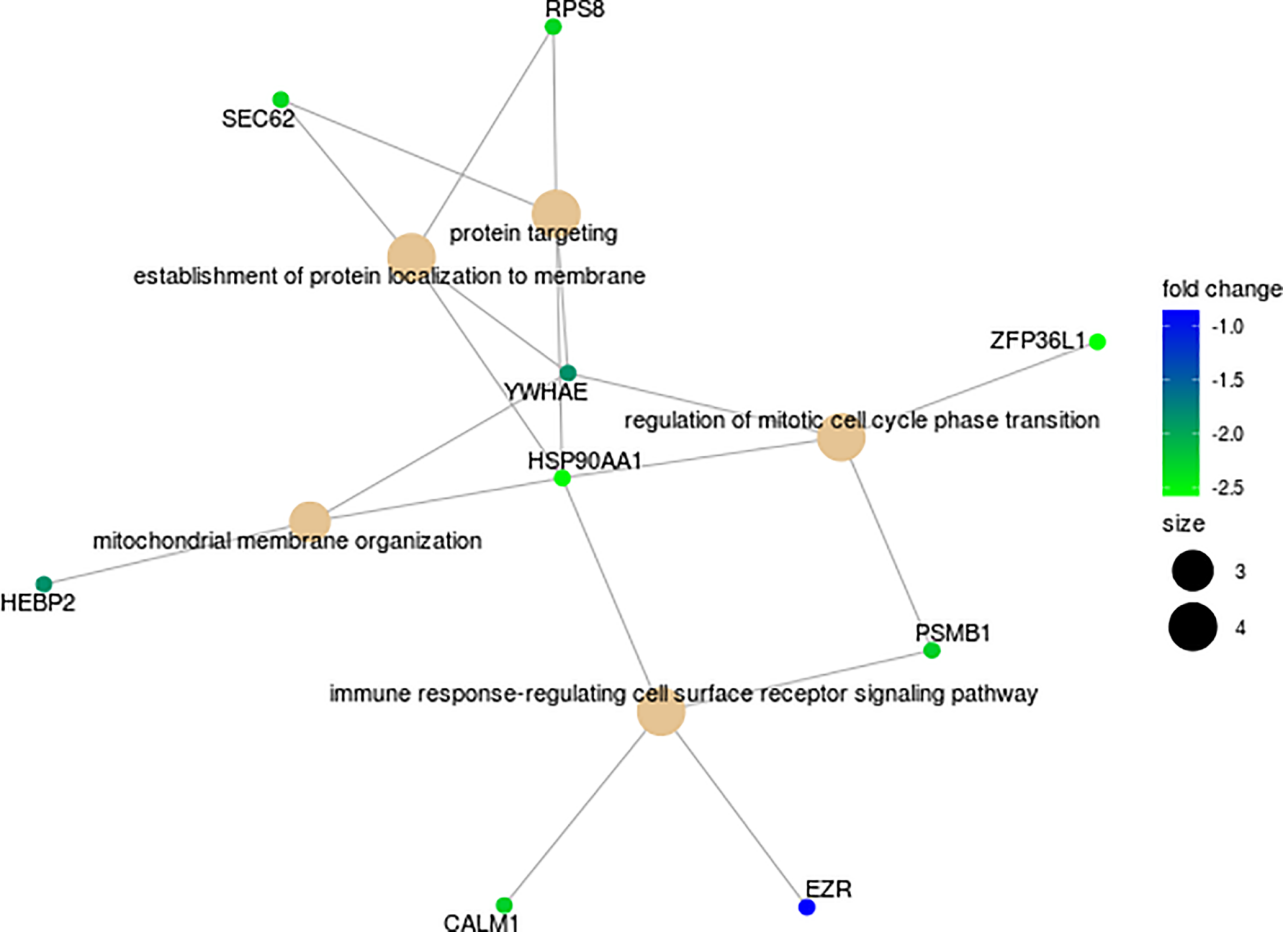
Functional enrichment map of 16 putative target DEGs for the miRNAs (MIR4278, MIR4422 and MIR4779) in cervical cancer stem-like cells from Non-Responders (n=10) patients compared to Responders (n=21). Associations among GO Biological processes are represented by their shared genes. Node size indicates the number of genes contained in the pathway, and color gradient shows the corresponding log2 fold change.

Analyses of the putative regulation of target genes for the four miRNAs overexpressed in NR (MIR4278, MIR4422, MIR4779, MIR1268B) returned a total of 411 interactions. We restricted the targets by selecting those with significant (p<0.05) negative correlation values. This selection revealed 16 genes with putative regulation by MIR4278, MIR4422 and MIR4779 (**Figure 5** **;** **Supplementary Table 5**). Gene ontology biological processes were enriched with cell cycle transition, immune response cell surface signaling, protein targeting/localization, and mitochondrial membrane organization pathways (**Figure 5** **).**

### Selection of Biomarkers

Based on the 1050 DEGs from the comparison between NR and R CCSC, we evaluated the prediction performance based on the partial area under the ROC curve statistic (pAUC; Pepe et al., 2013). The 21 transcripts with the highest pAUC (0.1) values and their cancer pathogenesis role are described in **Table 2**. Of these genes, six lncRNA (*AL360091.3, AL360175.1*, *AC025254.1, AC093801.1*, *AC092354.1* and *AC016722.1*); four pseudogenes (*RBM22P2, AC073324.1, OR1X1P, RPL7P52* and *MTND5P25*) and coding *SLC36A2* and *SDS* and have not previously been shown to be transcripts in cancer.

**Table 2:** Description of the 21 highest values of pAUC (partial area under the ROC curve) from the differential expression genes of cervical cancer stem-like cells in patients with responsiveness (n=21) and failure (n=10) to chemoradiotherapy (NR *vs* R).

Gene	Gene name	Log2FC	PADJ	GO Molecular function	Cancer pathogenesis
SNX2*	Sorting Nexin 2	-3.01	4.76E-03	Epidermal growth factor receptor binding	Silencing alters sensitivity to anticancer drugs targeted to c-Met/EGFR in lung cancer cells (27).
ILF2*	Interleukin enhancer-binding factor 2	-2.73	2.60E-05	DNA/RNA binding	Overexpression associated with poor prognosis in CC (28).
***PDCD10***	Programmed Cell Death 10	-2.45	8.32E-04	Protein binding	Downregulation associated with Akt signaling protein in glioblastoma (29); dual role in drug resistance (30).
***SCAND1***	SCAN domain-containing protein 1	-1.97	7.76E-05	DNA-binding transcription factor activity	Expression reduced in prostate cancer cells, suggesting a tumor suppressor role (31).
***REXO2***	RNA Exonuclease 2	-1.83	1.09E-02	Exonuclease activity	High expression levels correlated with high pathological grade and Gleason score in prostate cancer (32).
HNRNPA0*	Heterogeneous nuclear ribonucleoprotein A0	-1.71	3.86E-03	Nucleic acid binding	Activated by DNA damage checkpoint kinases and regulator of diverse cancer cells growth (33).
***AK6***	Adenylate Kinase 6	-0.94	6.94E-03	Nucleotide binding	Highly expressed in cancers and correlated with a worse prognosis (34).
***OPA3***	Outer Mitochondrial Membrane Lipid Metabolism Regulator OPA3	1.04	1.88E-02	Regulation of lipid metabolic process	High expression observed in pancreatic cancer tissues (35).
***SLC36A2***	Solute carrier family 36 member 2	1.25	3.54E-02	Amino acid transmembrane transporter	–
***SDS***	Serine Dehydratase	1.38	3.13E-02	L-serine ammonia-lyase activity	–
***CCR4***	C-C chemokine receptor type 4	1.57	2.38E-02	Chemokine binding	Lower mRNA levels in local immune microenvironment of normal cervix than in CC (36).
***AC073324.1***	Solute Carrier Family 19 Member 3 Pseudogene	1.67	4.23E-02	–	–
RBM22P2*	RNA Binding Motif Protein 22 Pseudogene 2	1.72	7.49E-03	–	–
***AL360091.3***	LncRNA	1.82	1.43E-02	–	–
AL360175.1*	LncRNA	2.05	1.90E-03	–	–
***AC025254.1***	LncRNA	2.10	4.34E-03	–	–
***OR1X1P***	Olfactory Receptor Family 1 Subfamily X Member 1 Pseudogene	2.13	3.76E-03	–	–
***AC093801.1***	LncRNA	2.20	1.90E-03	–	–
AC016722.1*	LncRNA	2.39	1.23E-03	–	–
***MTND5P25***	MT-ND5 Pseudogene 25	2.53	1.13E-03	–	–
AC092354.1*	LncRNA	2.59	8.53E-04	–	–

Log2 fold change (Log2FC) values represent the difference in expression observed in NR when compared to R. Transcripts are sorted by ascending number of Log2FC values. (PADJ, Adjusted p- value < 0.05; GO, Gene Ontology; CC, Cervical Cancer). The genes in common with AUC analysis are highlighted with “*”.

**Figure 6:**
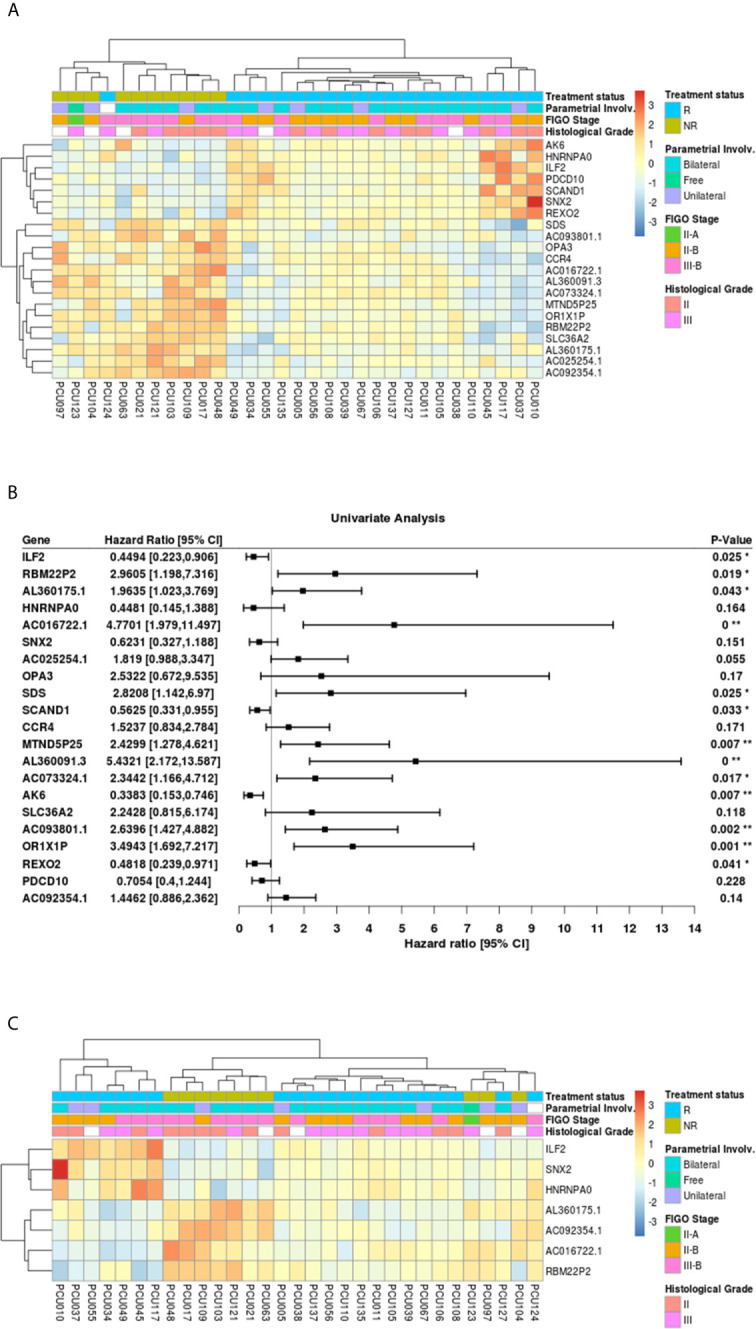
**(A)** Heatmap of the 21 genes with highest values of partial area under the ROC curve (pAUC, 0.1) from the 1050 differentially expressed transcripts (adjusted p-value<0.05) in cervical cancer stem-like cells. Responders patients (R, n=21) are represented in blue and Non-Responders (NR; n=10) are represented in green. Clinical features of Parametrial involvement, FIGO stages and Histological grades are detailed. **(B)** Forest plot for prognostic effect of the 21 highest pAUC differential expressed genes in cervical cancer stem-like cells using a univariate analysis. **(C)** Heatmap showing the 7 overlapping differential expressed loci (adjusted p-value <0.05) between pAUC (0.1) and AUC in cervical cancer stem-like cells. Responders patients (R) are represented in blue and Non-Responders (NR) are represented in green. Clinical characteristics of parametrial involvement, FIGO stages and Histological grades are detailed. (* < 0.05, ** <0.01 and 0** < 0.0005).

The 21-top ranked pAUC genes from the CCSC DEGs separated into two groups using unsupervised cluster analysis, reflecting the treatment status between NR and R patients (**Figure 6A**). The only exception was patient PCU124 who clustered with the NR group.

The Hazard ratios (HR, 95% CI) for the 21 highest pAUC genes under a univariate analysis are shown in **Figure 6B**. Of those, the expression of *ILF2* (HR=0.44, p=0.025), *SCAND1* (HR=0.56, p=0.033), *AK6* (HR=0.34, p=0.007) and *REXO2* (HR=0.48, p=0.041), demonstrated a protective effect on patient survival, while the genes *SDS* (HR=2.82, p=0.025), *OR1X1P* (HR=3.49, p=0.001), pseudogenes *RBM22P2* (HR=2.96, p=0.019), *MTND5P25* (HR=2.43, p=0.007), *AC073324.1* (HR=2.34, p=0.077), lncRNA *AC016722.1* (HR=4.77, p<0.0005), *AL360091.3* (HR=5.43, p<0.0005), *AC093801.1* (HR=2.64, p=0.002) and *AL360175.1* (HR=1.96, p=0.043), presented an increased risk with increased expression.

To refine the number of robust biomarkers with potentially diagnostic utility, we also identified the highest 22 AUC values from the 1050 DEGs (**Supplementary Table 6**). Then, selecting the common genes between the pAUC and AUC analysis we identified seven loci as potential biomarkers for distinguishing NR *vs.* R patients: *ILF2*, *SNX2*, *HNRNPA0*, *RBM22P2*, *ACO16722.1*, *AL360175.1* and *AC092354.1* (**Figure 6C**; **Tables 2**, **3**). Of these seven potential biomarkers for distinguishing NR *vs.* R patients, four also returned a significant HR for protection (*ILF2*) or for increased risk (*RBM22P2*, *ACO16722.1* and *AL360175.1).* To assess the quality parameters of prognostic prediction, accuracy, sensitivity, and specificity were evaluated using a univariate Logistic Regression (LR) for the seven loci (**Table 3**). All parameters showed predictive values higher than 70%. The genes *ILF2* and *RBM22P2* presented with the highest accuracy (93%) and specificity (100%); AL360175.1 had the best sensitivity (90%).

**Table 3:** Quality parameters of univariate Logistic Regression analysis (LR).

Gene	Accuracy	Sensitivity	Specificity	Gene Function	Log2FC
***SNX2***	0.90	0.70	1	Intracellular protein trafficking	-3.01
***ILF2***	0.93	0.80	1	Transcription factor for T-cell *IL2* expression	-2.73
***HNRNPA0***	0.90	0.80	0.95	Pre-mRNA processing, mRNA metabolism and transport	-1.71
***RBM22P2***	0.93	0.80	1	Autophagy and intracellular protein trafficking	-1.62
***AL360175.1***	0.90	0.90	0.90	LncRNA	2.05
***AC016722.1***	0.90	0.80	0.95	LncRNA	2.39
***AC092354.1***	0.90	0.80	0.95	LncRNA	2.59

Seven loci intersect between the highest 21 values of pAUC and AUC analysis from the differential expression genes in cervical cancer stem-like cells. Log2 fold change values (Log2FC, adjusted p-value <0.05) represent the difference in expression in Non-responders (n=10) compared to Responders (n=21) to the chemoradiotherapy.

Gene interaction network analysis identified ten proteins with the highest likelihood of functional relationships with *ILF2*: *HNRNPA1, HNRNPA2B1, HNRNPC, PTBP1, ERH, HNRNPH1, ILF3, CDC5L, HNRNPL* and *HNRNPR.* Amongst these, the first three genes are DEGs under expressed in NR.

## Discussion

Nearly half a million new cases of CC occur each year, with the majority of cases being diagnosed in developing countries and at advanced disease stages (1). In addition, the number of deaths from CC is expected to increase to 410,000 by 2030 (37). It is evident that CC continues to be an important public health challenge globally. Despite screening programs and the recent advent of HPV vaccines, the quality of screening and treatment options must be improved. Based on cancer stem cell research and deep sequencing approaches, molecular alterations in CC have been widely explored including tumor heterogeneity, which has brought new insights with the potential to impact clinical practice.

CSCs are self-renewing cancer cells responsible for expansion of the malignant mass in a dynamic process shaping the tumor microenvironment. CSCs have multiple differentiation capacities, anchorage independent growth, and chemoresistance (38). They represent approximately 0.1–10% of all tumor cells and express low levels of typical tumor-associated antigens compared to other tumor cells (39). After cytotoxic therapy regimens, residual tumor cells are enriched with CSCs, suggesting the importance of these cells in chemoresistance and disease relapse. CSCs may hijack host immune surveillance to escape the toxic effects of chemotherapy and evasion of apoptosis, resulting in typically aggressive tumors with poor prognosis (40). Despite representing a minimal number in the bulk tumor, CSCs actively coordinate intrinsic and extrinsic adaptation throughout carcinogenesis and therapy refractoriness. These unique properties place CSCs as a key component to be investigated, since their unique molecular signature might uncover effective therapeutic strategies in CC (41).

CD34 is a sialomucin family-related transmembrane protein that is involved in the modulation of cell adhesion and signal transduction (42). While it was first identified as a hallmark of hematopoietic stem cells, studies demonstrated that CD34 is also present on nonhematopoietic cell lines, including embryonic fibroblasts and vascular endothelial progenitors (43). Here we used CD34+ CD45- to sort subpopulations of tumor cells using flow cytometry as the most effective approach towards enriching stem-like tumor cells, thereby eliminating hematopoietic stem cells which are CD34+CD45+.

Based on the enriched CCSC from the bulk tumor biopsy, we conducted a low input RNA sequencing in patients with success (n=21) and refractoriness (n=10) to conventional chemoradiotherapy. The landscape of DEGs between the Non-responders (NR) and Responders (R) revealed 1050 genes (**Figure 2A**), characterized by coding, pseudogenes, long and small non-coding transcripts.

Interestingly, most of the coding DEGs from CCSC showed negative values of expression when comparing NR to R patients (**Figures 2A, B**). The wide-ranging pattern of under expression in CCSCs might suggest a state of dormancy/quiescence, a key feature of tumor plasticity that protects stem-like cells from antiproliferative chemotherapeutic agents (44). While CCSCs represent a key driving factor of tumorigenesis and metastasis, the majority maintain in a quiescent or dormant state until changes occur in the microenvironment (45). Two main mechanisms account for tumor resistance to classical therapeutic approaches: Darwinian selection of cells harboring novel genetic variations (extrinsic resistance), or epigenetic events (chromatin remodeling and activation of pathways to cell stress), where dormancy and/or tumor quiescence can be achieved (46, 47).

Gene Ontology (GO) analysis revealed enrichment of biomolecule synthesis GO terms, where pathways of DNA/RNA and protein processing, as well as cell metabolism are overrepresented by suppressed genes (**Figure 3**). This is consistent with previous studies that suggest regulatory gene networks are downregulated in quiescent stem cells in glioblastoma (48) and ovarian cancer (49).

Indeed, epigenetic adaptations are often reflected in CCSCs resistance capabilities. Non-coding elements in the genome hold a diversity of regulatory factors responsible for the expression of proto-oncogenes or tumor-suppressor genes (50). Given that the deregulation of small and lncRNAs are strongly implicated in the tumorigenesis of CC, we performed a thorough investigation of these RNAs in our dataset. Amongst the 24 DEGs reported (**Table 1** **;** **Supplementary Table 4**), the canonical oncogenic lncRNAs *MALAT1* (17, 18), *NEAT1 (* 20) and *NORAD* (24) showed negative expression values in NR as compared to R patients. Our finding differs from that seen in some of these studies, but it is noteworthy that we are comparing expression levels from purified CCSCs, which represented a small percentage of cells in the entire tumor mass. Interestingly, decreased expression of the oncosupressive lncRNA *GAS5* is correlated with tumor development and worse clinical outcome in CC patients (22, 23). These apparently opposing findings are not unexpected in cancer studies, especially due to the molecular heterogeneity of tumors, diverse sample sources (cervical tissues, cell exfoliates, mucus, serum, cell cultures, and purified cell populations) and the variety of methodologies used. In fact, non-coding RNA in CC are mainly evaluated from immortalized cell lines (HeLa, CaSki, SiHa) and cervical tissues (**Table 1**), and gene expression analyses were performed using non-cancerous cells and tissues as the reference. In our study we are using purified human biopsy derived CCSCs and comparing between NR *vs.* R groups.

Correlation analysis and binding prediction were conducted to investigate the relationship between the miRNAs from CCSCs transcriptome and their potential target genes. The miRNAs MIR4278, MIR4422 and MIR4779 have been reported to exert a role as tumor suppressors, induction of apoptosis, cell cycle arrest and in overall cancer survival (51–53). Sixteen genes, enriched for the functions of cell cycle transition, immune response cell surface signaling, protein targeting/localization, and mitochondrial membrane organization pathways showed negative correlations with overexpression of these miRNAs (**Figure 5** and **Supplementary Table 5**). Noteworthy, a subset of genes directly involved in translational machinery (*ZFP36L1, RSP8, SEC62*), proteolytic degradation of intracellular proteins (*PSMB1*) regulation of centrosome cycle and progression (*CALM1*), cell surface structure adhesion, migration and organization (*EZR*) and oxidative stress (*HEBP2*) are included in the potentially down-modulated genes (**Figure 5**).

The molecular mechanisms of non-coding RNAs in CC requires further characterization, particularly regarding the interplay between the diverse classes of RNAs and deregulation of metabolic pathways. Furthermore, the detection of these molecules in the serum of CC patients might lead to biosignatures of clinical relevance in non-invasive liquid biopsies (54).

The CSCs model and single-cell technologies have provided an opportunity to study a heterogeneous collection of cells with distinct genetic and phenotypic properties within tumors and investigate what roles they play in disease processes and therapeutic response (55). For CC, numerous markers have been associated with stemness properties of these cells (**Supplementary Table 1**), mainly regarding cellular self-renew and pluripotency maintenance features. When comparing rlog2 expression of stemness markers in NR and R samples, a statistical difference in three genes was observed (**Figure 4**). The overexpression of the canonical transcriptional factor *NANOG* is a well-known marker in CCSCs (56), and it shows an increased expression in the NR cells. Conversely, higher expression of tumor suppressors *CDKN2A* and *PTEN* were observed in R cells. *CDKN2A* (P16/INK4A) acts as critical regulators of stem cell functions and defines a worse prognosis in CC (57). The *PTEN* gene is involved in key mechanisms specific for CSCs, such as self-renewal, quiescence/cell cycle, Epithelial-to-Mesenchymal-Transition (EMT) and treatment refractoriness (58), with decreased expression associated with aggressive cancer phenotypes (59). These findings support a speculation that higher stemness features in CCSCs are associated with treatment failure. In addition to driving cells to an undifferentiated stage with high proliferative capacity, stemness can poise cells to enter a senescence/quiescent state to escape death and reach a state of reversible cell cycle arrest (60, 61).

Here we focused on a set of genes with the highest potential for classifying NR *vs.* R patients using a combination of pAUC and AUC analyses. The 21-gene signature selected using the pAUC analysis (**Table 2**) clearly separated the NR and R patients in a cluster analysis (**Figure 6A**). The transcriptional profile of a subgroup of these genes; *ILF2, SCAND1, AK6, REXO2, SDS, OR1X1P, RBM22P2, MTND5P25, AC073324.1, AC016722.1, AL360091.3* and *AC093801.1* indicate significant prognostic capacity for CC pathogenesis (**Figure 6B**). Choosing genes that overlapped between the pAUC and AUC analysis led to a collection of seven genes that strongly segregated the NR *vs.* R groups: *ILF2*, *SNX2*, *HNRNPA0*, *RBM22P2*, *ACO16722.1*, *AL360175.1* and *AC092354.1* (**Figure 6C** and **Table 3**). The parameters of accuracy, specificity and sensitivity highlights the strong potential of these molecules to predict failure or success to chemoradiotherapy (**Table 3**). Moreover, single gene or multi-gene signature assays can be used to measure specific molecular pathway perturbations that could guide therapeutic decisions in the future. Thus, our approach using high-throughput RNAseq, where thousands of individual molecules were investigated, offers an initial set of biomarkers with the potential for clinical use upon further validation.

The Interleukin enhancer-binding factor 2 gene (*ILF2*, NF45) forms a complex with ILF3 (NF90) involved in transcription regulation (62), mitosis (63), and DNA repair by nonhomologous end joining (64). *ILF2* acts as a tumor promoter, with overexpression associated with poor prognosis in CC, pancreatic ductal adenocarcinoma, non-small cell lung cancer and breast cancer (28, 65). However, our CCSC analysis revealed the opposite association with *ILF2* overexpressed in CCSCs from patients with no CC recurrence after the chemoradiotherapy (**Figure 6**). This pattern of higher expression related to better survival in CC patients is also seen in The Human Protein Atlas database (p=0.087; https://www.proteinatlas.org/ENSG00000143621-ILF2/pathology/cervical+cancer). Interestingly, from the ten proteins with the highest functional interactions with *ILF2*, network interaction analysis revealed three transcripts also under expressed in NR patients. The gene*s HNRNPA1, HNRNPA2B1* and *HNRNPC* are ubiquitously expressed heterogeneous nuclear ribonucleoproteins (hnRNPs) involved in mRNA metabolism, splicing and regulation of alternative splicing events. Indeed, these genes are associated with carcinogenesis and metastasis with a diverse set of tumor types (66, 67). The spliceosomal host machinery is especially important for the high-risk HPV life cycle and transformation in CC. The viral oncoproteins E6 and E7 are responsible to abrogate P53 and RB functions, with the prevalence of different E6 isoforms linked to lesion severity (68).

Overall, our findings point towards a highly elaborated mechanism of treatment refractoriness and metastasis in cancer: the plasticity of CSCs in regulating the inter-conversion between dormancy and proliferation. When compared to R patients, NR patient CCSCs tend to show a comprehensive decrease in gene expression, mostly related to transcriptional and translational processes. We speculate that therapy resistance in CC amongst these patients is mediated by molecular signatures of growth arrested in CCSCs, where a quiescent/slow cycling state could promote survival and disease recurrence.

In conclusion, we performed a comprehensive transcriptome analysis of CC stem-like cells enriched from fresh tumor biopsies. Despite numerous efforts, the discovery and establishment of new biomarkers for CC prognosis are lacking. Currently, overall prediction is mainly defined by clinical parameters, and our results bring novel insights to the field. First, the landscape of intrinsic resistance to conventional chemoradiotherapy revealed seven distinguishing genes as novel putative biomarkers for predicting response to therapy in CC patients, with four returning significant HRs. Second, we defined a distinct subset of non-coding transcripts in stem-like cells from CC, adding to our knowledge concerning the epigenetic factors driving treatment refractoriness. Third, the selected genes, in addition to standard clinical parameters, offer new insights towards prognostic assessment and therapeutic support in clinical practice. Importantly, further molecular characterization in a larger cohort of cervical cancer patients is required to validate our findings and possibly develop their use as clinically actionable biomarkers in the future.

## Data Availability Statement

The data used in this article is available at the NCBI’s BioProject site under the accession number: PRJNA705088.

## Ethics Statement

The studies involving human participants were reviewed and approved by Mario Penna Institutional Review Board #1.583.784. The patients/participants provided their written informed consent to participate in this study.

## Author Contributions

LZ performed the NGS, contributed with the experimental design and wrote the paper. CM performed the FACS experiments, PM contributed with patient recruitment, clinicopathological and survival analysis. LC contributed with patient recruitment and FACS experiments, WM contributed with the experimental design and together IS and RD performed the bioinformatics analysis, LB contributed with paper writing, AT-C and OF participated in FACS experimental design and analysis. TF and SP were responsible for patient recruitment and clinicopathological data acquisition. KG was responsible for the study design, data analysis and paper writing, PS performed the anatomopathological analysis and contributed to experimental design and writing. All authors contributed to the article and approved the submitted version.

## Funding

This study was funded by the Ministry of Health (Pronon - Grant number: NUP:25000.159953/2014-18 and NUP:25000.079266/2015-09) and the Minas Gerais State Research Agency (FAPEMIG - Grant number: TCT 19.011-13 - RT 00003-13). KG is a CNPq Research Fellow.

## Conflict of Interest

The authors declare that the research was conducted in the absence of any commercial or financial relationships that could be construed as a potential conflict of interest.

## Abbreviations

CC, cervical cancer; CSC, cancer stem cell; CCSC, cervical cancer stem-like cells; CCNSC, Cancer non-stem cell; FACS, Fluorescence-activated cell sorting; DEG, Differentially expressed gene; Log2FC, Log2 fold change; AUC, Area under the curve; pAUC, partial area under the curve; ROC, Receiver operating characteristic curve; GLOBOCAN, Global Cancer Observatory; HPV, Human papillomavirus; RNA, Ribonucleotide; SCC, Squamous cell carcinoma; ADC, Adenocarcinoma; FIGO, International Federation of Gynecology and Obstetrics; FDR, False Discovery Rate; NR, Non-Responder; R, Responder; LR, Logistic Regression.
