# Supplementary figures and images for: Cervical Cancer Stem-Like Cell Transcriptome Profiles Predict Response to Chemoradiotherapy
Source: Front Oncol. 2021 May 7;11:639339. doi: 10.3389/fonc.2021.639339 (PMC8138064; doi:10.3389/fonc.2021.639339)

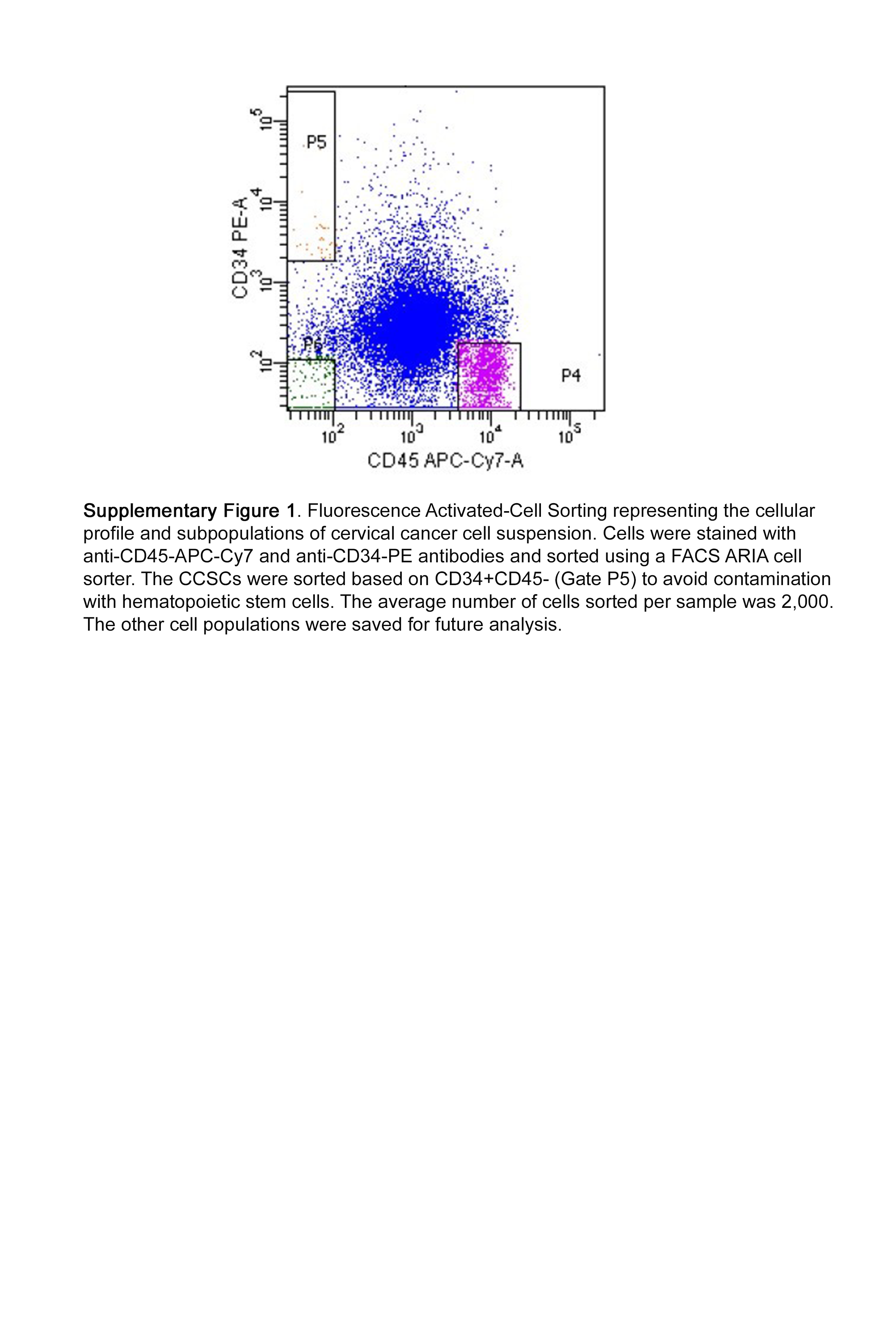

Supplement: Supplementary file 1 [file Image_1.tif]

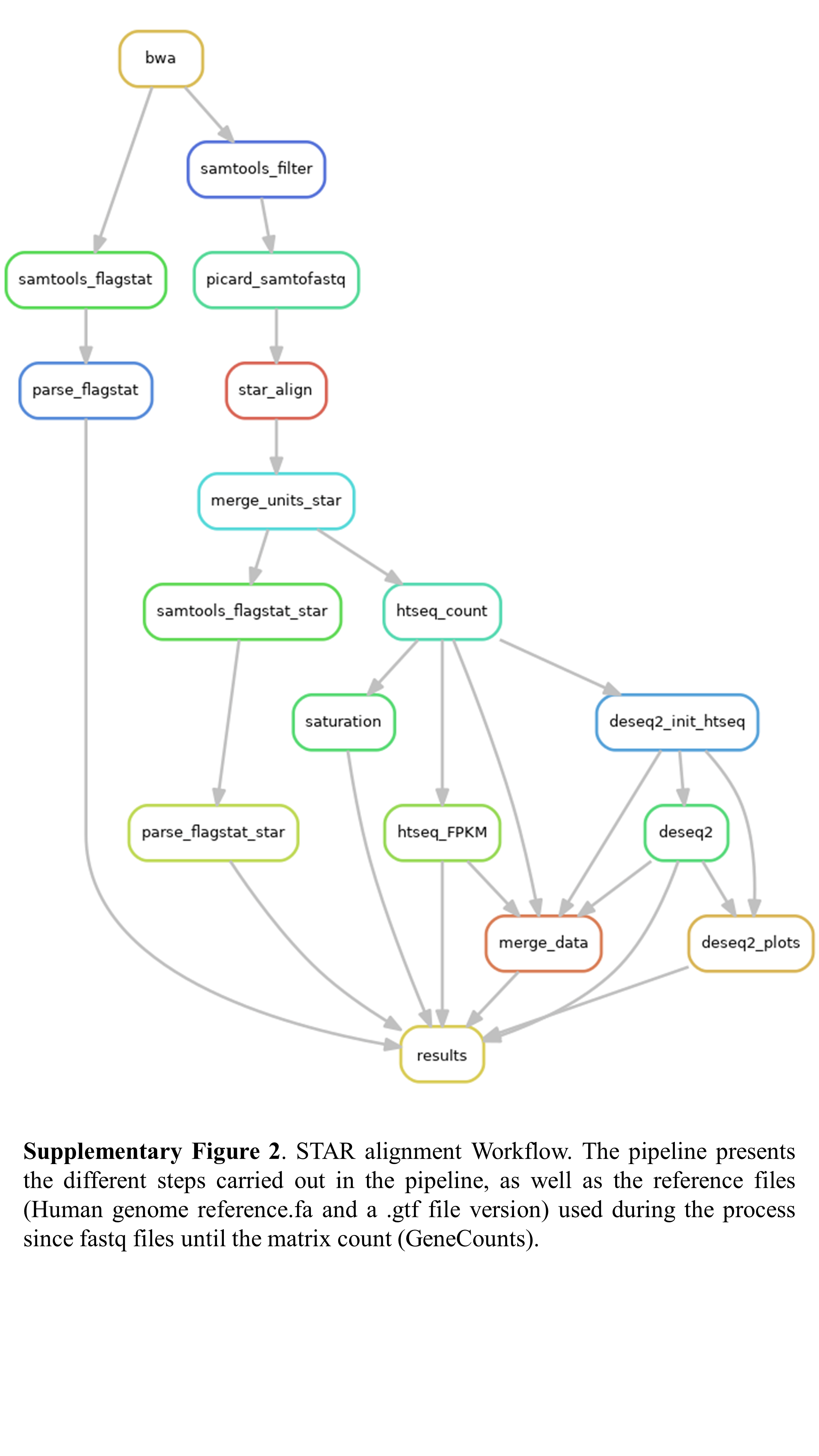

Supplement: Supplementary file 2 [file Image_2.tif]

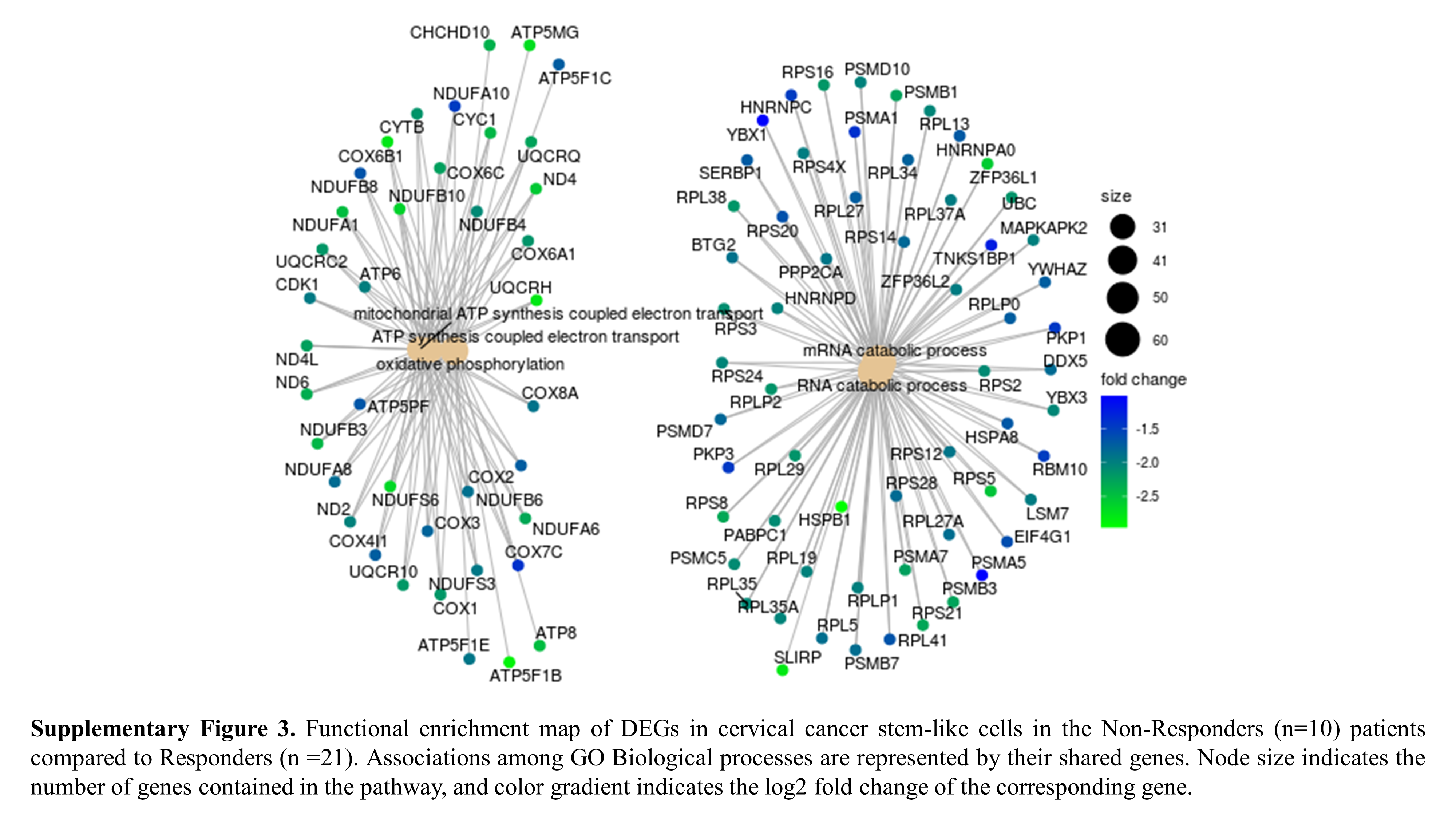

Supplement: Supplementary file 3 [file Image_3.tif]
