# Supplementary material for: Cervical Cancer Stem-Like Cell Transcriptome Profiles Predict Response to Chemoradiotherapy
Source: Front Oncol. 2021 May 7;11:639339. doi: 10.3389/fonc.2021.639339 (PMC8138064; doi:10.3389/fonc.2021.639339)
Supplement: Supplementary file 4 [file Table_1.docx]

**Supplementary Table 1.** Genes potentially related to stemness features in cervical cancer.

| **Transcript ID** | **Gene** | **Reference** |
| --- | --- | --- |
| ENSG00000085563 | *ABCB1* | 1 |
| ENSG00000103222 | *ABCC1* | 1 |
| ENSG00000118777 | *ABCG2* | 1 |
| ENSG00000075624 | *ACTB* | 2 |
| ENSG00000130402 | *ACTN4* | 3 |
| ENSG00000165092 | *ALDH1A1* | 1, 4 |
| ENSG00000138613 | *APH1B* | 5 |
| ENSG00000166710 | *B2M* | 2 |
| ENSG00000171791 | *BCL2* | 6 |
| ENSG00000026508 | *CD44* | 1, 6 |
| ENSG00000147889 | *CDKN2A (INK4)* | 7 |
| ENSG00000165140 | *FBP1* | 8 |
| ENSG00000149557 | *FEZ1* | 9 |
| ENSG00000111640 | *GAPDH* | 2 |
| ENSG00000165704 | *HPRT1* | 2 |
| ENSG00000091409 | *ITGA6* | 1 |
| ENSG00000096968 | *JAK2* | 10 |
| ENSG00000177606 | *JUN* | 11 |
| ENSG00000136826 | *KLF4* | 12 |
| ENSG00000128422 | *KRT17* | 12 |
| ENSG00000171345 | *KRT19* | 13 |
| ENSG00000139292 | *LGR5* | 14 |
| ENSG00000251562 | *MALAT1* | 15 |
| ENSG00000147649 | *MTDH* | 16 |
| ENSG00000111704 | *NANOG* | 12 |
| ENSG00000143217 | *NECTIN4* | 17 |
| ENSG00000116044 | *NFE2L2 (NRF2)* | 18 |
| ENSG00000148400 | *NOTCH1* | 19 |
| ENSG00000134250 | *NOTCH2* | 19 |
| ENSG00000074181 | *NOTCH3* | 19 |
| ENSG00000204301 | *NOTCH4* | 19 |
| ENSG00000169297 | *NR0B1 (DAX1)* | 20 |
| ENSG00000145623 | *OSMR* | 21 |
| ENSG00000125207 | *PIWIL1* | 22 |
| ENSG00000067225 | *PKM2* | 23 |
| ENSG00000204531 | *OCT3/4 (POU5F1)* | 1,4,24 |
| ENSG00000007062 | *PROM1* | 1 |
| ENSG00000148334 | *PTGES2* | 25 |
| ENSG00000079313 | *REXO1* | 26 |
| ENSG00000080345 | *RIF1* | 28 |
| ENSG00000164690 | *SHH* | 29 |
| ENSG00000181449 | *SOX2* | 1,24,27 |
| ENSG00000118785 | *SPP1* | 30 |
| ENSG00000168610 | *STAT3* | 10,31 |
| ENSG00000112592 | *TBP* | 2 |
| ENSG00000135111 | *TBX3* | 32 |
| ENSG00000072274 | *TFRC (CD71)* | 33 |
| ENSG00000136869 | *TLR4* | 34 |
| ENSG00000103671 | *TRIP4* | 35 |
| ENSG00000253352 | *TUG1* | 36 |
| ENSG00000141510 | *TP53* | 37 |
| ENSG00000171862 | *PTEN* | 38 |

1. Organista-Nava J et al. Cervical cancer stem cell-associated genes: Prognostic implications in cervical cancer. Oncol Lett. 2019 Jul;18(1):7-14.
2. de Campos RP et al. Cervical cancer stem-like cells: systematic review and identification of reference genes for gene expression. Cell Biol Int. 2018 Feb;42(2):139-152.
3. Jung J et al. α-Actinin-4 regulates cancer stem cell properties and chemoresistance in cervical cancer. Carcinogenesis. 2019 Oct 4. pii: bgz168.
4. Tulake W, Yuemaier R, Sheng L, Ru M, Lidifu D, Abudula A. Upregulation of stem cell markers ALDH1A1 and OCT4 as potential biomarkers for the early detection of cervical carcinoma. Oncol Lett. 2018 Nov;16(5):5525-5534.
5. Yang S et al. High-Risk Human Papillomavirus E7 Maintains Stemness Via APH1B In Cervical Cancer Stem-Cell Like Cells. Cancer Manag Res. 2019 Nov 12;11:9541-9552.
6. Zhang J et al. CD44+/CD24+-Expressing Cervical Cancer Cells and Radioresistant Cervical Cancer Cells Exhibit Cancer Stem Cell Characteristics. Gynecol Obstet Invest. 2019;84(2):174-182.
7. Fu HC et al. Low P16(INK4A) Expression Associated with High Expression of Cancer Stem Cell Markers Predicts Poor Prognosis in Cervical Cancer after Radiotherapy. Int J Mol Sci. 2018 Aug 27;19(9). pii: E2541.
8. Li H et al. Fructose‑1,6‑bisphosphatase‑1 decrease may promote carcinogenesis and chemoresistance in cervical cancer. Mol Med Rep. 2017 Dec;16(6):8563-8571.
9. Lan Y et al. FEZF1 is an Independent Predictive Factor for Recurrence and Promotes Cell Proliferation and Migration in Cervical Cancer. J Cancer. 2018 Oct 10;9(21):3929-3938.
10. Morgan EL, Macdonald A. JAK2 Inhibition Impairs Proliferation and Sensitises Cervical Cancer Cells to Cisplatin-Induced Cell Death. Cancers (Basel). 2019 Dec 4;11(12). pii: E1934.
11. yagi A et al. Cervical cancer stem cells manifest radioresistance: Association with upregulated AP-1 activity. Sci Rep. 2017 Jul 6;7(1):4781.
12. Bigoni-Ordóñez GD et al. Molecular iodine inhibits the expression of stemness markers on cancer stem-like cells of established cell lines derived from cervical cancer. BMC Cancer. 2018 Sep 26;18(1):928.
13. Mehrpouya M et al. Evaluation of cytokeratin 19 as a prognostic tumoral and metastatic marker with focus on improved detection methods. J Cell Physiol. 2019 Dec;234(12):21425-21435.
14. Cao HZ et al. LGR5 promotes cancer stem cell traits and chemoresistance in cervical cancer. Cell Death Dis. 2017 Sep 7;8(9):e3039.
15. Wang N et al. MALAT1 promotes cisplatin resistance in cervical cancer by activating the PI3K/AKT pathway. Eur Rev Med Pharmacol Sci. 2018 Nov;22(22):7653-7659.
16. Liu X et al. Knockdown of astrocyte elevated gene-1 (AEG-1) in cervical cancer cells decreases their invasiveness, epithelial to mesenchymal transition, and chemoresistance. Cell Cycle. 2014;13(11):1702-7.
17. Nayak A et al. Nanoquinacrine sensitizes 5-FU-resistant cervical cancer stem-like cells by down-regulating Nectin-4 via ADAM-17 mediated NOTCH deregulation. Cell Oncol (Dordr). 2019 Apr;42(2):157-171.
18. Jia Y et al. Aberrantly elevated redox sensing factor Nrf2 promotes cancer stem cell survival via enhanced transcriptional regulation of ABCG2 and Bcl-2/Bmi-1 genes. Oncol Rep. 2015 Nov;34(5):2296-304.
19. Rodrigues C et al. Notch signalling in cervical cancer. Exp Cell Res. 2019 Dec 15;385(2):111682.
20. Liu XF et al. DAX1 promotes cervical cancer cell growth and tumorigenicity through activation of Wnt/β-catenin pathway via GSK3β. Cell Death Dis. 2018 Mar 1;9(3):339.
21. Kucia-Tran JA et al. Overexpression of the oncostatin-M receptor in cervical squamous cell carcinoma is associated with epithelial-mesenchymal transition and poor overall survival. Br J Cancer. 2016 Jul 12;115(2):212-22.
22. Liu W et al. Hiwi facilitates chemoresistance as a cancer stem cell marker in cervical cancer. Oncol Rep. 2014 Nov;32(5):1853-60.
23. Lin Y et al. Knockdown of PKM2 enhances radiosensitivity of cervical cancer cells. Cancer Cell Int. 2019 May 14;19:129.
24. Rachmadi L et al. Role of Cancer Stem Cell, Apoptotic Factor, DNA Repair, and Telomerase Toward Radiation Therapy Response in Stage IIIB Cervical Cancer. Oman Med J. 2019 May;34(3):224-230.
25. Kuroda H et al. Prostaglandin E2 produced by myeloid-derived suppressive cells induces cancer stem cells in uterine cervical cancer. Oncotarget. 2018 Nov 20;9(91):36317-36330.
26. Zeng YT et al. REX1 promotes EMT-induced cell metastasis by activating the JAK2/STAT3-signaling pathway by targeting SOCS1 in cervical cancer. Oncogene. 2019 Oct;38(43):6940-6957.
27. Yang WT et al. NF-YA transcriptionally activates the expression of SOX2 in cervical cancer stem cells. PLoS One. 2019 Jul 31;14(7):e0215494.
28. Mei Y et al. Silencing RIF1 decreases cell growth, migration and increases cisplatin sensitivity of human cervical cancer cells. Oncotarget. 2017 Nov 6;8(63):107044-107051.
29. Sharma A et al. Sonic hedgehog pathway activation regulates cervical cancer stem cell characteristics during epithelial to mesenchymal transition. J Cell Physiol. 2019 Feb 4.
30. Chen X et al. SPP1 inhibition improves the cisplatin chemo-sensitivity of cervical cancer cell lines. Cancer Chemother Pharmacol. 2019 Apr;83(4):603-613.
31. Shukla S et al. Level of phospho-STAT3 (Tyr705) correlates with copy number and physical state of human papillomavirus 16 genome in cervical precancer and cancer lesions. PLoS One. 2019 Sep 5;14(9):e0222089.
32. Dong L et al. The special stemness functions of Tbx3 in stem cells and cancer development. Semin Cancer Biol. 2019 Aug;57:105-110.
33. Leung TH et al. CD71+ Population Enriched by HPV-E6 Protein Promotes Cancer Aggressiveness and Radioresistance in Cervical Cancer Cells. Mol Cancer Res. 2019 Sep;17(9):1867-1880.
34. Bahramabadi R et al. TLR4: An Important Molecule Participating in Either Anti-Human Papillomavirus Immune Responses or Development of Its Related Cancers. Viral Immunol. 2019 Dec;32(10):417-423.
35. Che Y et al. TRIP4 promotes tumor growth and metastasis and regulates radiosensitivity of cervical cancer by activating MAPK, PI3K/AKT, and hTERT signaling. Cancer Lett. 2019 Jun 28;452:1-1.
36. Wei X et al. Low expression of TUG1 promotes cisplatin sensitivity in cervical cancer by activating the MAPK pathway. J BUON. 2019 May-Jun;24(3):1020-1026.
37. Ghatak D et al. Cancer Stemness: p53 at the Wheel. Front Oncol. 2021 Jan 11;10:604124.
38. Kim RJ et al. PTEN loss-mediated Akt activation increases the properties of cancer stem-like cell populations in prostate cancer. Oncology. 2014;87(5):270-9.

**Supplementary Table 2.** Clinicopathological characteristics and chemoradiotherapy response.

| **Patient characteristics** | | **N** | **Value** |
| --- | --- | --- | --- |
| Age | Years | 31 | 52.3±16.8 |
| Diagnostic | SCC | 30 | 96.8 |
|  | ICA | 1 | 3.2 |
| FIGO Stage | IIA | 1 | 3.2 |
|  | IIB | 15 | 48.4 |
|  | IIIB | 15 | 48.4 |
| Histological grade | II | 13 | 41.9 |
|  | III | 13 | 41.9 |
|  | NA | 5 | 16.1 |
| Parametrial involvement | Free | 1 | 3.2 |
|  | Unilaterally | 7 | 22.6 |
|  | Bilaterally | 22 | 71.0 |
|  | NA | 1 | 3.2 |
| Vaginal involvement | Yes | 28 | 90.3 |
|  | No | 1 | 3.2 |
|  | NA | 2 | 6.5 |
| Lymph node status | NX | 8 | 25.8 |
|  | N0 | 9 | 29.0 |
|  | N1 | 14 | 45.2 |
| Metastasis | MX | 10 | 32.3 |
|  | M0 | 9 | 29.0 |
|  | M1 | 12 | 38.7 |
| Tumor size (cm) | <4 | 1 | 3.2 |
|  | ≥4 | 29 | 93.5 |
|  | NA | 1 | 3.2 |
| Overall response | R | 21 | 67.7 |
|  | NR | 10 | 32.3 |

The values represent the mean + standard error or %. SCC, Squamous Cell Carcinoma; ADC, Adenocarcinoma; NR, Non-Responders, R, Responders; NX, Regional lymph nodes cannot be assessed; N0, No regional lymph node metastasis; N1, Regional lymph node metastasis; MX, Distant metastasis cannot be assessed; M0, No distant metastasis; M1, Distant metastasis. NA, Not Available.

**Supplementary Table 3.** Gene Ontology pathways.

| **Pathway** | **pval** | **padj** | **NES** | **Size** |
| --- | --- | --- | --- | --- |
| RNA_Binding | 9.49E-23 | 6.83E-20 | -3.71 | 216 |
| Cellular_Macromolecule_Localization | 1.40E-22 | 6.83E-20 | -3.84 | 192 |
| Intracellular_Transport | 1.79E-21 | 5.82E-19 | -3.75 | 183 |
| Mitochondrial_Envelope | 1.18E-19 | 2.88E-17 | -3.78 | 128 |
| Mitochondrion | 6.81E-19 | 1.33E-16 | -3.53 | 197 |
| Envelope | 8.74E-18 | 1.22E-15 | -3.57 | 162 |
| Symbiotic_Process | 3.08E-17 | 3.76E-15 | -3.61 | 151 |
| Macromolecule_Catabolic_Process | 5.22E-17 | 5.67E-15 | -3.50 | 162 |
| Protein_Containing_Complex_Subunit_Organization | 8.11E-17 | 7.92E-15 | -3.40 | 195 |
| Apoptotic_Process | 3.20E-16 | 2.84E-14 | -3.41 | 181 |
| Organonitrogen_Compound_Biosynthetic_Process | 3.91E-16 | 3.18E-14 | -3.40 | 176 |
| Establishment_Of_Protein_Localization_To_Organelle | 4.43E-16 | 3.33E-14 | -3.68 | 87 |
| Structural_Molecule_Activity | 5.29E-16 | 3.69E-14 | -3.61 | 105 |
| Protein_Localization_To_Membrane | 9.11E-16 | 5.93E-14 | -3.65 | 85 |
| mRNA_Metabolic_Process | 1.94E-15 | 1.18E-13 | -3.42 | 140 |
| Ribonucleoprotein_Complex | 3.23E-15 | 1.86E-13 | -3.43 | 133 |
| Ion_Transport | 4.23E-15 | 2.30E-13 | -3.49 | 97 |
| Cellular_Macromolecule_Catabolic_Process | 1.11E-14 | 5.72E-13 | -3.39 | 151 |
| Catalytic_Complex | 1.98E-14 | 9.68E-13 | -3.31 | 141 |
| Response_To_Biotic_Stimulus | 4.73E-14 | 2.01E-12 | -3.36 | 131 |
| Regulation_Of_Cell_Death | 5.02E-14 | 2.04E-12 | -3.25 | 161 |
| Anchoring_Junction | 8.89E-14 | 3.39E-12 | -3.44 | 94 |
| Regulation_Of_Cell_Population_Proliferation | 3.53E-13 | 1.15E-11 | -3.30 | 122 |
| Transmembrane_Transport | 4.21E-13 | 1.33E-11 | -3.25 | 115 |
| Response_To_Oxygen_Containing_Compound | 4.39E-13 | 1.34E-11 | -3.23 | 126 |
| Regulation_Of_Organelle_Organization | 1.11E-12 | 3.09E-11 | -3.26 | 111 |
| Cell_Motility | 1.39E-12 | 3.76E-11 | -3.19 | 115 |
| Response_To_Cytokine | 1.63E-12 | 4.31E-11 | -3.18 | 115 |
| Regulation_Of_Response_To_Stress | 2.44E-12 | 5.95E-11 | -3.15 | 115 |
| Cell_Cycle | 4.25E-12 | 9.67E-11 | -3.12 | 140 |
| Membrane_Protein_Complex | 6.22E-12 | 1.38E-10 | -3.20 | 109 |
| Epithelium_Development | 6.97E-12 | 1.51E-10 | -3.27 | 108 |
| Epithelial_Cell_Differentiation | 8.12E-12 | 1.69E-10 | -3.34 | 68 |
| Cellular_Amide_Metabolic_Process | 1.51E-11 | 3.07E-10 | -3.09 | 136 |
| Response_To_Endogenous_Stimulus | 1.69E-11 | 3.37E-10 | -3.14 | 119 |
| Negative_Regulation_Of_Response_To_Stimulus | 2.12E-11 | 4.14E-10 | -3.07 | 128 |
| Response_To_Abiotic_Stimulus | 3.58E-11 | 6.85E-10 | -3.15 | 105 |
| Secretion | 3.66E-11 | 6.87E-10 | -3.08 | 120 |
| Nuclear_Transcribed_MRNA_Catabolic_Process | 3.77E-11 | 6.95E-10 | -3.34 | 51 |
| Positive_Regulation_Of_Signaling | 5.21E-11 | 9.26E-10 | -3.07 | 127 |
| Negative_Regulation_Of_Biosynthetic_Process | 5.61E-11 | 9.79E-10 | -3.09 | 118 |
| Positive_Regulation_Of_Molecular_Function | 7.41E-11 | 1.23E-09 | -3.06 | 129 |
| Regulation_Of_Intracellular_Signal_Transduction | 9.24E-11 | 1.51E-09 | -3.03 | 137 |
| Whole_Membrane | 1.23E-10 | 1.97E-09 | -3.04 | 125 |
| Regulation_Of_Transport | 1.25E-10 | 1.98E-09 | -3.09 | 105 |
| Identical_Protein_Binding | 1.69E-10 | 2.49E-09 | -2.95 | 145 |
| Cell_Activation | 1.67E-10 | 2.49E-09 | -3.00 | 133 |
| Positive_Regulation_Of_Protein_Metabolic_Process | 1.72E-10 | 2.51E-09 | -3.01 | 137 |
| Positive_Regulation_Of_Cellular_Biosynthetic_Process | 2.33E-10 | 3.26E-09 | -2.94 | 144 |
| Membrane_Organization | 2.47E-10 | 3.40E-09 | -3.04 | 98 |
| Proteolysis | 2.65E-10 | 3.59E-09 | -2.99 | 137 |
| Keratinocyte_Differentiation | 2.74E-10 | 3.64E-09 | -3.28 | 33 |
| Regulation_Of_Protein_Modification_Process | 3.18E-10 | 3.98E-09 | -2.97 | 133 |
| Generation_Of_Precursor_Metabolites_And_Energy | 3.87E-10 | 4.73E-09 | -3.08 | 79 |
| Response_To_Nitrogen_Compound | 4.20E-10 | 5.06E-09 | -3.13 | 76 |
| Defense_Response | 5.87E-10 | 6.82E-09 | -2.97 | 127 |
| Negative_Regulation_Of_Nucleobase_Containing_  Compound_Metabolic_Process | 6.09E-10 | 7.00E-09 | -3.02 | 113 |
| Mitotic_Cell_Cycle | 6.26E-10 | 7.11E-09 | -3.01 | 93 |
| Protein_Containing_Complex_Binding | 6.34E-10 | 7.12E-09 | -2.99 | 106 |
| Epidermis_Development | 7.31E-10 | 8.11E-09 | -3.17 | 50 |
| Homeostatic_Process | 1.45E-09 | 1.50E-08 | -2.85 | 126 |
| Biological_Adhesion | 1.54E-09 | 1.58E-08 | -2.97 | 93 |
| Response_To_Inorganic_Substance | 1.55E-09 | 1.58E-08 | -3.09 | 53 |
| Exocytosis | 1.85E-09 | 1.82E-08 | -2.97 | 91 |
| Regulation_Of_Transferase_Activity | 2.03E-09 | 1.95E-08 | -3.00 | 88 |
| Oxidation_Reduction_Process | 2.38E-09 | 2.25E-08 | -2.96 | 100 |
| Blood_Vessel_Morphogenesis | 2.44E-09 | 2.29E-08 | -3.11 | 36 |
| Positive_Regulation_Of_Cellular_Component_Organization | 2.71E-09 | 2.50E-08 | -2.95 | 91 |
| Positive_Regulation_Of_Nucleobase_Containing_Compound_  Metabolic_Process | 3.11E-09 | 2.78E-08 | -2.88 | 137 |
| Regulation_Of_Cellular_Component_Movement | 3.37E-09 | 3.00E-08 | -3.04 | 60 |
| Cell_Cell_Signaling | 3.83E-09 | 3.37E-08 | -2.90 | 99 |
| Endoplasmic_Reticulum | 5.06E-09 | 4.41E-08 | -2.96 | 90 |
| Enzyme_Regulator_Activity | 5.46E-09 | 4.72E-08 | -2.96 | 73 |
| Ribonucleotide_Binding | 5.54E-09 | 4.75E-08 | -2.89 | 93 |
| Cytoskeleton_Organization | 5.91E-09 | 4.92E-08 | -2.91 | 91 |
| Regulation_Of_Proteolysis | 5.95E-09 | 4.92E-08 | -2.98 | 64 |
| Regulation_Of_Catabolic_Process | 6.89E-09 | 5.65E-08 | -2.87 | 97 |
| Regulation_Of_Phosphorus_Metabolic_Process | 7.10E-09 | 5.78E-08 | -2.83 | 120 |
| Regulation_Of_Cell_Differentiation | 7.28E-09 | 5.88E-08 | -2.81 | 111 |
| Tube_Development | 8.47E-09 | 6.67E-08 | -2.97 | 58 |
| Regulation_Of_Hydrolase_Activity | 2.33E-08 | 1.70E-07 | -2.84 | 91 |
| Negative_Regulation_Of_Molecular_Function | 2.97E-08 | 2.04E-07 | -2.91 | 82 |
| Posttranscriptional_Regulation_Of_Gene_Expression | 3.05E-08 | 2.08E-07 | -2.86 | 83 |
| Regulation_Of_Cellular_Localization | 3.28E-08 | 2.23E-07 | -2.88 | 67 |
| Signaling_Receptor_Binding | 3.47E-08 | 2.34E-07 | -2.72 | 103 |
| Positive_Regulation_Of_Multicellular_Organismal_Process | 4.09E-08 | 2.72E-07 | -2.79 | 104 |
| Ubiquitin_Like_Protein_Ligase_Binding | 4.70E-08 | 3.10E-07 | -2.97 | 52 |
| Negative_Regulation_Of_Protein_Metabolic_Process | 5.91E-08 | 3.85E-07 | -2.77 | 85 |
| Ribonucleoprotein_Complex_Biogenesis | 6.10E-08 | 3.95E-07 | -2.85 | 60 |
| Chromosome_Organization | 7.34E-08 | 4.66E-07 | -2.75 | 85 |
| Anatomical_Structure_Formation_Involved_In_Morphogenesis | 9.70E-08 | 5.96E-07 | -2.82 | 70 |
| Ameboidal_Type_Cell_Migration | 9.88E-08 | 5.99E-07 | -2.94 | 31 |
| Response_To_Lipid | 1.12E-07 | 6.56E-07 | -2.81 | 70 |
| Synapse | 1.33E-07 | 7.67E-07 | -2.77 | 81 |
| Cellular_Response_To_DNA_Damage_Stimulus | 2.12E-07 | 1.17E-06 | -2.73 | 74 |
| Positive_Regulation_Of_Developmental_Process | 2.41E-07 | 1.31E-06 | -2.73 | 81 |
| DNA_Metabolic_Process | 2.64E-07 | 1.41E-06 | -2.76 | 68 |
| Response_To_Organic_Cyclic_Compound | 3.71E-07 | 1.94E-06 | -2.72 | 73 |
| Endothelial_Cell_Migration | 4.35E-07 | 2.24E-06 | -2.76 | 17 |
| Supramolecular_Complex | 4.43E-07 | 2.25E-06 | -2.63 | 84 |
| Nuclear_Outer_Membrane_Endoplasmic_Reticulum_  Membrane_Network | 4.45E-07 | 2.25E-06 | -2.67 | 72 |
| Immune_System_Development | 4.52E-07 | 2.28E-06 | -2.68 | 80 |
| Signal_Transduction_By_Protein_Phosphorylation | 4.63E-07 | 2.31E-06 | -2.73 | 75 |
| Hydrolase_Activity_Acting_On_Acid_Anhydrides | 5.10E-07 | 2.53E-06 | -2.76 | 63 |
| Negative_Regulation_Of_Cell_Adhesion | 5.51E-07 | 2.67E-06 | -2.80 | 20 |
| Nuclear_Body | 5.64E-07 | 2.69E-06 | -2.70 | 68 |
| Protein_Modification_By_Small_Protein_Conjugation_Or_  Removal | 5.80E-07 | 2.74E-06 | -2.62 | 84 |
| Growth | 7.35E-07 | 3.44E-06 | -2.72 | 62 |
| Response_To_Growth_Factor | 8.36E-07 | 3.87E-06 | -2.66 | 55 |
| Regulation_Of_Cellular_Component_Biogenesis | 8.49E-07 | 3.89E-06 | -2.68 | 67 |
| Regulation_Of_Peptidase_Activity | 9.29E-07 | 4.24E-06 | -2.79 | 41 |
| Central_Nervous_System_Development | 9.53E-07 | 4.33E-06 | -2.75 | 54 |
| Epithelial_Cell_Proliferation | 1.01E-06 | 4.57E-06 | -2.77 | 31 |
| Regulation_Of_Immune_System_Process | 1.09E-06 | 4.88E-06 | -2.58 | 112 |
| Kinase_Binding | 1.10E-06 | 4.91E-06 | -2.69 | 62 |
| Transcription_Factor_Binding | 1.28E-06 | 5.61E-06 | -2.65 | 60 |
| Response_To_Oxidative_Stress | 1.45E-06 | 6.24E-06 | -2.68 | 47 |
| Negative_Regulation_Of_Multicellular_Organismal_Process | 1.71E-06 | 7.29E-06 | -2.67 | 63 |
| Microtubule_Cytoskeleton | 1.88E-06 | 8.01E-06 | -2.66 | 66 |
| Cadherin_Binding | 2.26E-06 | 9.38E-06 | -2.60 | 57 |
| Vacuole | 2.75E-06 | 1.11E-05 | -2.59 | 70 |
| Perinuclear_Region_Of_Cytoplasm | 2.83E-06 | 1.13E-05 | -2.57 | 55 |
| Cell_Projection_Organization | 2.87E-06 | 1.14E-05 | -2.56 | 81 |
| Lipid_Metabolic_Process | 3.01E-06 | 1.19E-05 | -2.59 | 71 |
| Plasma_Membrane_Region | 3.17E-06 | 1.24E-05 | -2.57 | 61 |
| Nucleolus | 4.01E-06 | 1.54E-05 | -2.53 | 77 |
| Lipid_Binding | 4.06E-06 | 1.54E-05 | -2.68 | 37 |
| Cytoskeletal_Protein_Binding | 4.14E-06 | 1.56E-05 | -2.56 | 61 |
| Enzyme_Linked_Receptor_Protein_Signaling_Pathway | 4.67E-06 | 1.75E-05 | -2.55 | 68 |
| Response_To_Endoplasmic_Reticulum_Stress | 5.13E-06 | 1.91E-05 | -2.58 | 25 |
| Neuron_Projection | 5.43E-06 | 1.99E-05 | -2.52 | 69 |
| Embryo_Development | 7.03E-06 | 2.51E-05 | -2.52 | 71 |
| Animal_Organ_Morphogenesis | 7.50E-06 | 2.66E-05 | -2.53 | 62 |
| Response_To_Toxic_Substance | 7.93E-06 | 2.81E-05 | -2.61 | 28 |
| Pigment_Granule | 8.32E-06 | 2.94E-05 | -2.54 | 23 |
| Neurogenesis | 8.75E-06 | 3.04E-05 | -2.48 | 81 |
| Intrinsic_Component_Of_Plasma_Membrane | 1.20E-05 | 4.11E-05 | -2.47 | 68 |
| Extracellular_Matrix | 1.34E-05 | 4.53E-05 | -2.55 | 35 |
| Organelle_Assembly | 1.38E-05 | 4.64E-05 | -2.50 | 66 |
| Reproduction | 1.63E-05 | 5.42E-05 | -2.42 | 86 |
| Nucleoside_Triphosphate_Metabolic_Process | 1.68E-05 | 5.54E-05 | -2.51 | 16 |
| Cell_Junction_Organization | 1.73E-05 | 5.66E-05 | -2.55 | 49 |
| Reproductive_System_Development | 2.18E-05 | 7.06E-05 | -2.54 | 42 |
| Peptidyl_Amino_Acid_Modification | 2.21E-05 | 7.14E-05 | -2.46 | 63 |
| Organophosphate_Metabolic_Process | 2.23E-05 | 7.17E-05 | -2.40 | 65 |
| Regulation_Of_Anatomical_Structure_Morphogenesis | 2.41E-05 | 7.67E-05 | -2.41 | 69 |
| Golgi_Apparatus | 2.44E-05 | 7.72E-05 | -2.37 | 101 |
| Protein_Domain_Specific_Binding | 2.52E-05 | 7.92E-05 | -2.51 | 56 |
| Transition_Metal_Ion_Binding | 2.57E-05 | 8.02E-05 | -2.42 | 61 |
| Organelle_Localization | 2.58E-05 | 8.02E-05 | -2.46 | 48 |
| Organic_Acid_Metabolic_Process | 3.08E-05 | 9.41E-05 | -2.38 | 67 |
| Microtubule_Based_Process | 3.41E-05 | 1.04E-04 | -2.45 | 47 |
| Carbohydrate_Derivative_Metabolic_Process | 3.43E-05 | 1.04E-04 | -2.37 | 64 |
| Protein_Dimerization_Activity | 3.46E-05 | 1.04E-04 | -2.38 | 69 |
| Response_To_Wounding | 4.96E-05 | 1.42E-04 | -2.44 | 50 |
| Actin_Cytoskeleton | 7.27E-05 | 2.02E-04 | -2.44 | 37 |
| Cytokine_Production | 7.63E-05 | 2.10E-04 | -2.27 | 59 |
| Reactive_Oxygen_Species_Metabolic_Process | 1.11E-04 | 2.94E-04 | -2.36 | 29 |
| Process_Utilizing_Autophagic_Mechanism | 1.22E-04 | 3.17E-04 | -2.41 | 44 |
| Ossification | 1.34E-04 | 3.42E-04 | -2.24 | 26 |
| Maintenance_Of_Location_In_Cell | 2.28E-04 | 5.28E-04 | -2.29 | 20 |
| Calcium_Dependent_Protein_Binding | 2.98E-04 | 6.60E-04 | -2.26 | 17 |
| Aging | 3.19E-04 | 6.93E-04 | -2.26 | 30 |
| Muscle_Structure_Development | 4.27E-04 | 8.87E-04 | -2.27 | 46 |
| Regulation_Of_Body_Fluid_Levels | 4.30E-04 | 8.90E-04 | -2.22 | 29 |
| Membrane_Region | 4.65E-04 | 9.45E-04 | -2.27 | 32 |
| Receptor_Mediated_Endocytosis | 5.55E-04 | 1.08E-03 | -2.17 | 23 |
| Nuclear_Envelope | 9.35E-04 | 1.69E-03 | -2.13 | 38 |
| Protein_Folding | 9.48E-04 | 1.71E-03 | -2.13 | 23 |
| Protein_Maturation | 2.82E-03 | 4.34E-03 | -2.02 | 21 |
| Hydrolase_Activity_Acting_On_Ester_Bonds | 3.44E-03 | 5.08E-03 | -1.86 | 26 |

**Supplementary Table 4.** Long non-coding RNAs (lncRNA) and small RNA (miRNA*) differentially expressed in cervical cancer stem-like cells from Non-responders (NR) and Responders (R) related to cancer pathogenesis. Based on lncRNAs and small RNAs from public databases and literature, 15 transcripts associated with cancer tumorigenesis were detected. Log2 fold change (Log2FC) values represent the difference in expression observed in NR when compared to R. (PADJ: Adjusted p-value <0.05; CC: cervical cancer).

| **Gene** | **Log2FC** | **PADJ** | **Role in cancer** | **Reference** |
| --- | --- | --- | --- | --- |
| ***PDCD4-AS1*** | -1.89 | 4.44E-02 | Controls breast cancer progression by promoting tumor suppressor gene mRNA stability | 1 |
| ***MAGI1-IT1*** | -1.59 | 3.73E-02 | Promotes invasion and metastasis of epithelial ovarian cancer | 2 |
| ***PRECSIT*** | -1.43 | 3.56E-02 | Promotes progression of cutaneous squamous cell carcinoma | 3 |
| ***LINC00470*** | 1.36 | 3.45E-02 | Promotes proliferation and invasion in glioma and hepatocellular carcinoma; attenuates chemosensitivity in glioma; oncogenic functions on gastric cancer cell | 4-6 |
| ***LINC00974*** | 1.49 | 3.51E-02 | Promotes cell cycle progression in gastric carcinoma and proliferation a metastasis in hepatocellular carcinoma | 7,8 |
| ***LINC00449*** | 1.47 | 4.47E-02 | Regulates the proliferation and invasion of acute monocytic leukemia | 9 |
| ***MZF1-AS1*** | 1.50 | 2.78E-02 | Induces the proline synthesis, tumorigenesis, and aggressiveness of neuroblastoma cells | 10 |
| ***AC010789.1*** | 1.80 | 1.37E-02 | Correlates with the prognosis of patients with colorectal cancer | 11 |
| ***MIR1268B**** | 1.82 | 4.22E-02 | Confers chemosensitivity in breast cancer; detected in plasma of lung cancer patients | 12,13 |
| ***MIR4779**** | 1.86 | 2.41E-02 | Suppresses tumor growth by inducing apoptosis and cell cycle arrest | 14 |
| ***AC079341.1*** | 1.89 | 8.80E-03 | Associated with the progression and prognosis of stage I hepatocellular cancer | 15 |
| ***LINC01048*** | 1.94 | 2.26E-02 | High expression is an unfavorable prognostic factor for patients with squamous cell carcinoma | 16 |
| ***MIR4278**** | 2.00 | 3.22E-02 | Suppressor and antagonist of oncogenic MAPK in Burkitt lymphoma; upregulated expression in gastric cancer | 17,18 |
| ***MIR4422**** | 2.30 | 1.14E-02 | Associated with survival outcome in non-small cell lung cancer | 19 |
| ***AC012640.3*** | 2.42 | 7.00E-04 | Expression can predict survival in hepatocellular carcinoma | 20 |

- - - 1. Jadaliha M, et al. A natural antisense lncRNA controls breast cancer progression by promoting tumor suppressor gene mRNA stability. PLoS Genet. 2018 Nov 29;14(11):e1007802.
      2. Gao H, et al. Long noncoding RNA MAGI1-IT1 promoted invasion and metastasis of epithelial ovarian cancer via the miR-200a/ZEB axis. Cell Cycle. 2019 Jun;18(12):1393-1406.
      3. Piipponen M, et al. p53-Regulated Long Noncoding RNA PRECSIT Promotes Progression of Cutaneous Squamous Cell Carcinoma via STAT3 Signaling. Am J Pathol. 2020 Feb;190(2):503-517. doi: 10.1016/j.ajpath.2019.10.019. Epub 2019 Dec 12. Erratum in: Am J Pathol. 2020 Apr;190(4):916
      4. Wu C, et al. LINC00470 promotes tumour proliferation and invasion, and attenuates chemosensitivity through the LINC00470/miR-134/Myc/ABCC1 axis in glioma. J Cell Mol Med. 2020 Sep 11.
      5. Huang W, et al. LncRNA LINC00470 promotes proliferation through association with NF45/NF90 complex in hepatocellular carcinoma. Hum Cell. 2020 Jan;33(1):131-139.
      6. Yan J, et al. LncRNA LINC00470 promotes the degradation of PTEN mRNA to facilitate malignant behavior in gastric cancer cells. Biochem Biophys Res Commun. 2020 Jan 22;521(4):887-893.
      7. Gao H, et al. LncRNA LINC00974 Upregulates CDK6 to Promote Cell Cycle Progression in Gastric Carcinoma. Cancer Biother Radiopharm. 2019 Dec;34(10):666-670.
      8. Tang J, et al. A novel biomarker Linc00974 interacting with KRT19 promotes proliferation and metastasis in hepatocellular carcinoma. Cell Death Dis. 2014 Dec 4;5(12):e1549.
      9. Shi Y, et al. LINC00449 regulates the proliferation and invasion of acute monocytic leukemia and predicts favorable prognosis. J Cell Physiol. 2020 Oct;235(10):6536-6547.
      10. Fang E, et al. Therapeutic Targeting of MZF1-AS1/PARP1/E2F1 Axis Inhibits Proline Synthesis and Neuroblastoma Progression. Adv Sci (Weinh). 2019 Aug 10;6(19):1900581.
      11. Wang X et al. A 15-lncRNA signature predicts survival and functions as a ceRNA in patients with colorectal cancer. Cancer Manag Res. 2018 Nov 16;10:5799-5806.
      12. Zhu WJ et.al. MiR-1268b confers chemosensitivity in breast cancer by targeting ERBB2-mediated PI3K-AKT pathway. Oncotarget. 2017 Aug 9;8(52):89631-89642.
      13. Asakura et.al. A miRNA-based diagnostic model predicts resectable lung cancer in humans with high accuracy. Commun Biol. 2020 Mar 19;3(1):134.
      14. Koo KH, Kwon H. MicroRNA miR-4779 suppresses tumor growth by inducing apoptosis and cell cycle arrest through direct targeting of PAK2 and CCND3. Cell Death Dis. 2018 Jan 23;9(2):77.
      15. Gu X et al. Construction and Comprehensive Analyses of a Competing Endogenous RNA Network in Tumor-Node-Metastasis Stage I Hepatocellular Carcinoma. Biomed Res Int. 2020 Feb 11;2020:5831064.
      16. Chen L et al. USF1-induced upregulation of LINC01048 promotes cell proliferation and apoptosis in cutaneous squamous cell carcinoma by binding to TAF15 to transcriptionally activate YAP1. Cell Death Dis. 2019 Apr 1;10(4):296.
      17. Wang W et al. MicroRNA-4728 serves as a suppressor and antagonist of oncogenic MAPK in Burkitt lymphoma. Saudi J Biol Sci. 2018 Jul;25(5):982-985.
      18. Liu D et al. Identification of Aberrantly Expressed miRNAs in Gastric Cancer. Gastroenterol Res Pract. 2014;2014:473817.
      19. Zhao Y et al. Polymorphisms in MicroRNAs are associated with survival in non-small cell lung cancer. Cancer Epidemiol Biomarkers Prev. 2014 Nov;23(11):2503-11.
      20. Ye J, et al. Risk scoring based on expression of long non‑coding RNAs can effectively predict survival in hepatocellular carcinoma patients with or without fibrosis. Oncol Rep. 2020 May;43(5):1451-1466.

**Supplementary Table 5.** miRNA and putative targets differentially expressed in cervical cancer stem-like cells with opposite regulation among patients with responsiveness (Responder, n=21) and failure (Non-Responder, n=10) in the chemoradiotherapy. Log2 fold change (Log2FC) values represent the difference in expression observed in Non-responders compared to Responders. (PADJ: Adjusted p-value<0.05).

| **miRNA** | **Log2FC** | **PADJ** | **Target Gene Symbol** | **Log2FC** | **PADJ** | **NR correlation** | **Correlation p-value** |
| --- | --- | --- | --- | --- | --- | --- | --- |
| MIR4278 | 2.00 | 3.22E-02 | *C1GALT1C1* | -1.68 | 4.06E-02 | -0.78 | 7.55E-03 |
|  |  |  | *YIF1A* | -1.83 | 1.72E-02 | -0.78 | 7.59E-03 |
|  |  |  | *MRPL44* | -2.06 | 1.01E-02 | -0.73 | 1.64E-02 |
|  |  |  | *SAP30L* | -1.90 | 6.67E-03 | -0.73 | 1.76E-02 |
|  |  |  | *EFHD2* | -1.83 | 2.81E-02 | -0.70 | 2.44E-02 |
|  |  |  | *CCDC25* | -1.84 | 1.25E-02 | -0.70 | 2.55E-02 |
|  |  |  | *YWHAE* | -1.84 | 1.68E-02 | -0.63 | 4.89E-02 |
| MIR4422 | 2.30 | 1.14E-02 | *ZFP36L1* | -2.57 | 1.95E-02 | -0.87 | 1.07E-03 |
|  |  |  | *HSP90AA1* | -2.53 | 1.73E-03 | -0.87 | 1.21E-03 |
|  |  |  | *CALM1* | -2.26 | 4.64E-03 | -0.78 | 7.34E-03 |
|  |  |  | *SEC62* | -2.31 | 5.97E-04 | -0.78 | 8.27E-03 |
|  |  |  | *CHIC2* | -1.71 | 1.88E-02 | -0.74 | 1.39E-02 |
|  |  |  | *RPS8* | -2.30 | 5.55E-03 | -0.67 | 3.47E-02 |
|  |  |  | *PSMB1* | -2.26 | 6.90E-03 | -0.67 | 3.50E-02 |
| MIR4779 | 1.86 | 2.41E-02 | *EZR* | -0.86 | 1.19E-02 | -0.75 | 1.30E-02 |
|  |  |  | *HEBP2* | -1.84 | 1.07E-02 | -0.66 | 3.63E-02 |

**Supplementary Table 6.** Description of the 22 highest values of AUC (area under the ROC curve) from the differential expression genes of cervical cancer stem-like cells in patients with responsiveness (Responder, n=21) and failure (Non-Responder, n=10) in the chemoradiotherapy. Log2 fold change (Log2FC) values represent the difference in expression observed in Non-responders compared to Responders. Transcripts are sorted by ascending number of Log2FC. (PADJ: Adjusted p- value < 0.05; LncRNA: long non-coding RNA; TEC: To be Experimental Tested).

| **Transcript ID** | **Gene** | **Gene name** | **Log2FG** | **PADJ** |
| --- | --- | --- | --- | --- |
| ENSG00000251340 | ***MTCYBP35*** | Mitochondrially encoded Cytochrome B Pseudogene 35 | -4.08 | 2.08E-03 |
| ENSG00000205302 | ***SNX2*** | Sorting Nexin 2 | -3.01 | 4.76E-03 |
| ENSG00000178974 | ***FBXO34*** | F-Box Protein 34 | -2.89 | 1.00E-02 |
| ENSG00000143621 | ***ILF2*** | Interleukin enhancer-binding factor 2 | -2.73 | 2.60E-05 |
| ENSG00000100028 | ***SNRPD3*** | Small Nuclear Ribonucleoprotein D3 Polypeptide | -2.64 | 7.51E-05 |
| ENSG00000090520 | ***DNAJB11*** | DNAJ Heat Shock Protein Family (Hsp40) Member B11 | -2.15 | 5.30E-04 |
| ENSG00000125871 | ***MGME1*** | Mitochondrial Genome Maintenance Exonuclease 1 | -1.91 | 5.87E-03 |
| ENSG00000177733 | ***HNRNPA0*** | Heterogeneous Nuclear Ribonucleoprotein A0 | -1.71 | 3.86E-03 |
| ENSG00000111481 | ***COPZ1*** | Coatomer subunit zeta-1 | -1.62 | 6.45E-03 |
| ENSG00000133773 | ***CCDC59*** | Coiled-Coil Domain Containing 59 | -1,62 | 9,55E-03 |
| ENSG00000116786 | ***PLEKHM2*** | Pleckstrin Homology RUN Domain Containing M2 | -1.37 | 7.03E-03 |
| ENSG00000251629 | ***LINC02241*** | LncRNA | 1.23 | 1.38E-02 |
| ENSG00000178229 | ***ZNF543*** | Zinc Finger Protein 543 | 1.43 | 6.90E-03 |
| ENSG00000131095 | ***GFAP*** | Glial Fibrillary Acidic Protein | 1.58 | 1.01E-02 |
| ENSG00000279632 | ***AP003108.4*** | TEC | 1.61 | 2.37E-02 |
| ENSG00000228697 | ***AL023755.1*** | LncRNA | 1.64 | 4.25E-03 |
| ENSG00000213411 | ***RBM22P2*** | RNA Binding Motif Protein 22 Pseudogene 2 | 1.72 | 7.49E-03 |
| ENSG00000280422 | ***AC115284.2*** | TEC | 1.75 | 2.39E-02 |
| ENSG00000265561 | ***MIR1268B*** | MicroRNA | 1.82 | 4.22E-02 |
| ENSG00000233470 | ***AL360175.1*** | LncRNA | 2.05 | 1.90E-03 |
| ENSG00000226548 | ***AC016722.1*** | LncRNA | 2.39 | 1.23E-03 |
| ENSG00000272354 | ***AC092354.1*** | LncRNA | 2.59 | 8.53E-04 |
